# Evolution of Multidrug Resistance during *Staphylococcus aureus* Infection Involves Mutation of the Essential Two Component Regulator WalKR

**DOI:** 10.1371/journal.ppat.1002359

**Published:** 2011-11-10

**Authors:** Benjamin P. Howden, Christopher R. E. McEvoy, David L. Allen, Kyra Chua, Wei Gao, Paul F. Harrison, Jan Bell, Geoffrey Coombs, Vicki Bennett-Wood, Jessica L. Porter, Roy Robins-Browne, John K. Davies, Torsten Seemann, Timothy P. Stinear

**Affiliations:** 1 Department of Microbiology and Immunology, University of Melbourne, Victoria, Australia; 2 Austin Centre for Infection Research (ACIR), Infectious Diseases Department, Austin Health, Heidelberg, Victoria, Australia; 3 Microbiology Department, Austin Health, Heidelberg, Victoria, Australia; 4 Department of Microbiology, Monash University, Clayton, Victoria, Australia; 5 Victorian Bioinformatics Consortium, Monash University, Clayton, Victoria, Australia; 6 SA Pathology, Womens and Children’s Hospital, Adelaide, South Australia, Australia; 7 Microbiology Department, Royal Perth Hospital, Perth, Western Australia, Australia; National Institute of Allergy and Infectious Diseases, National Institutes of Health, United States of America

## Abstract

Antimicrobial resistance in *Staphylococcus aureus* is a major public health threat, compounded by emergence of strains with resistance to vancomycin and daptomycin, both last line antimicrobials. Here we have performed high throughput DNA sequencing and comparative genomics for five clinical pairs of vancomycin-susceptible (VSSA) and vancomycin-intermediate ST239 *S. aureus* (VISA); each pair isolated before and after vancomycin treatment failure. These comparisons revealed a frequent pattern of mutation among the VISA strains within the essential *walKR* two-component regulatory locus involved in control of cell wall metabolism. We then conducted bi-directional allelic exchange experiments in our clinical VSSA and VISA strains and showed that single nucleotide substitutions within either *walK* or *walR* lead to co-resistance to vancomycin and daptomycin, and caused the typical cell wall thickening observed in resistant clinical isolates. Ion Torrent genome sequencing confirmed no additional regulatory mutations had been introduced into either the *walR* or *walK* VISA mutants during the allelic exchange process. However, two potential compensatory mutations were detected within putative transport genes for the *walK* mutant. The minimal genetic changes in either *walK* or *walR* also attenuated virulence, reduced biofilm formation, and led to consistent transcriptional changes that suggest an important role for this regulator in control of central metabolism. This study highlights the dramatic impacts of single mutations that arise during persistent *S. aureus* infections and demonstrates the role played by *walKR* to increase drug resistance, control metabolism and alter the virulence potential of this pathogen.

## Author Summary

The treatment of serious infections caused by *Staphylococcus aureus* is complicated by the development of antibiotic resistance, and recently resistance to one of the last available antibiotics to treat resistant *S. aureus* infections, vancomycin, has also emerged. Here we have shown using whole genome sequencing of 10 *S. aureus* strains and gene replacement experiments on sequential *S. aureus* isolates obtained during persistent bloodstream infection, how *S. aureus* evolved intermediate vancomycin resistance by acquiring mutations in the important regulator WalKR. Mutations in this locus were found to be common in strains of *S. aureus* demonstrating intermediate vancomycin resistance, and these strains also demonstrated daptomycin non-susceptibility even though this drug had never been used for treatment. Experiments to replace the mutated *walK* or *walR* into the parent strain and vice versa confirmed that these mutations were responsible for the antibiotic resistance, but also led to significant changes in virulence, biofilm formation, and regulation of metabolism within the organism. This study highlights the adaptability of *S. aureus* in the face of antimicrobial treatment.

## Introduction

In hospitals worldwide infections with methicillin-resistant *S. aureus* (MRSA) remain a significant cause of morbidity and mortality, with a small number of clones accounting for a large number of hospital acquired infections. In Australasia, multi-locus sequence type (MLST) 239 (ST239) is the major hospital acquired clone of MRSA, and has been present in the region for over 30 years. This clone is resistant to almost all antibiotic classes; therefore the mainstay of therapy for serious MRSA infections has been the glycopeptide antibiotic vancomycin. However, resistant strains have recently emerged [1], and although the level of resistance is low there is an impact on treatment outcome [2]. These vancomycin-intermediate *S. aureus* (VISA, vancomycin MIC 4–8 µg/ml) and heterogenous-VISA (hVISA, vancomycin MIC ≤2 µg/ml with a “resistant subpopulation”) strains are increasingly common, however the genetics of resistance are incompletely defined [3]. While the emergence of VISA in Australia has been in strains of the ST239 clone [4], the first VISA strain Mu50 was reported from Japan in 1997 [5], and a number of other reported VISA strains belong to the same clonal complex as Mu50 (CC5) [6]–[8].

In many cases VISA emerge from fully-vancomycin susceptible *S. aureus* (VSSA) parental strains during persistent infection [6], [8], [9], and in some cases this has been associated with the evolution of daptomycin non-susceptibility despite the absence of exposure to daptomycin [10]. VISA strains appear to arise via sequential point mutations in key staphylococcal regulatory genes [11]–[13], however the breadth of mutations that can contribute to resistance are poorly defined. In addition, it is not clear if there are differences in resistance mechanisms and pathways to VISA in different clones of *S. aureus*. Commonly described phenotypic changes in VISA compared to VSSA include increased cell wall thickness and reduced autolytic activity [7], [14], [15], in addition to other significant phenotypic changes that are predicted to impact the virulence of the organism. These include a reduction in biofilm formation, reduced activity of the *agr* quorum sensing system, and enhanced capsule production [3], [4], [15], [16]. The link(s) between development of antimicrobial resistance and the regulation of these virulence factors is unknown.

A number of studies have used comparative genomics of paired *S. aureus* isolates to detect mutations that occur in the resistant strain compared to the parent strain, including a landmark study by Mwangi *et al* where increasing vancomycin resistance in sequential clinical isolates of *S. aureus* were linked to accumulated mutations in the increasingly resistant strain [11]. However, the genetic loci where mutations in clinical *S. aureus* strains have been experimentally confirmed using allelic replacement experiments to contribute to VISA are limited to *vraSR*, *graRS,* and more recently *rpoB* [13], [17], [18]. We have previously used functional genomics to show that a point mutation in *graS* can lead to reduced vancomycin susceptibility in one clinical pair of ST239 VSSA (JKD6009) and VISA (JKD6008) [18]. However this mutation, while leading to a reduction in vancomycin susceptibility, did not restore the full VISA resistance profile. It is worth noting that all these studies have focussed on a total of three clinical and laboratory induced VISA isolates, and screening of additional ST239 VISA strains has failed to demonstrate that mutations in these loci are common to other VISA [4], suggesting that there are mutations in as yet undefined loci contributing to VISA in other clinical isolates.

Daptomycin is an antibiotic that exerts its effect at the cell membrane, and while a link between VISA and daptomycin non-susceptibility has been demonstrated [10], [19], the genetics of this relationship are undefined. While mutations in *mprF*, *rpoB* and *rpoC* are thought to be the genetic basis for daptomycin non-susceptibility in *S. aureus*, mutations have also been detected in *walK* [20]. The contribution of the *walK* mutations to daptomycin non-susceptibility has not been defined as these strains also harboured *mprF* mutations.

To expand understanding of the mechanisms of VISA and identify other loci contributing to vancomycin resistance we fully sequenced four additional clinical pairs of VSSA and VISA, and then compared them to the fully assembled and annotated genome of our previously described VISA strain JKD6008 [21]. We also re-analyzed our original sequenced pair (JKD6008 and JKD6009) after fully assembling the genome sequence of the VISA strain JKD6008. Using this approach, followed by allelic replacement experiments, we show that WalKR (also known as YycGF and VicKR) plays a major role during the *in vivo* evolution of extensive drug resistance in clinical *S. aureus*. Our findings highlight an unexpected degree of plasticity within this essential two-component regulator and are particularly pertinent, with inhibitors of WalK recently proposed as novel anti-staphylococcal agents [22]–[24].

## Results

### Isolate Selection and Characteristics

To identify the genetic mechanisms leading to VISA five clinical pairs of VSSA and VISA were selected, where the resistant strain evolved from the susceptible parent strain during failed vancomycin therapy [15], [18]. In addition, we also examined eight global non-paired hVISA and VISA (Table 1). The clinical pairs were selected from patients that had been treated with vancomycin, and not daptomycin. This meant that any changes in daptomycin susceptibility linked to vancomycin exposure and increasing vancomycin resistance could be specifically assessed. The duration of *in vivo* vancomycin exposure ranged from 8 to 42 days in these clinical isolate pairs and the majority of isolates fulfilled the criteria for VISA (vancomycin broth MIC 4–8 µg per ml). For all VISA strains in the isolate pairs an increase in daptomycin MIC was seen compared to the parental VSSA strain, and five of the 13 hVISA/VISA isolates were daptomycin non-susceptible (Table 1). All of the clinical strains were typed using the StaphyType96 DNA Array (CLONDIAG, Jena, Germany) to predict the sequence type, *mec* type, and *agr* type (Table 1). All of the clinical pairs were from Australia and New Zealand, and were ST239 MRSA, the dominant hospital clone of MRSA in the region, while the additional isolates represented a range of staphylococcal sequence types within clonal complexes 5 and 8.

**Table 1:** Methicillin-resistant *Staphylococcus aureus* clinical strains used in study.

Strain	Origin	Clonal Complex	Typing ST- *mec*	*agr* type	Vanco BMD (µg/mL)	Dapto Etest MIC (µg/mL)	Etest GRD vanco 48hs (µg/mL)	Etest GRD teico 48hs (µg/mL)	Resistance Phenotype	Drug Exposure^a^	Reference or Source
**Isolate Pairs**											
***Pair 1***											
JKD6000	Australia	8	ST239-III [3A]	*agr*_I	1	0.25	1.5	4	VSSA, DS		[4], [15]
JKD6001		8	ST239-III [3A]	*agr*_I	4	1.5	4	12	VISA, DNS	Vanc 13 d	[4], [15]
***Pair 2***											
JKD6004	Australia	8	ST239-III [3A]	*agr*_I	2	0.5	1.5	2	VSSA, DS		[4], [15]
JKD6005		8	ST239-III [3A]	*agr*_I	4	2	3	32	VISA, DNS	Vanc 8 d	[4], [15]
***Pair 3***											
JKD6009	New Zealand	8	ST239-III [3A]	*agr*_I	2	0.19	1.5	3	VSSA, DS		[4], [15]
JKD6008		8	ST239-III [3A]	*agr*_I	4	0.5	3	32	VISA, DS	Vanc 42 d	[4], [15]
***Pair 4***											
JKD6021	Australia	8	ST239-III [3A]	*agr*_I	1	0.19	1.5	4	VSSA, DS		[4], [15]
JKD6023		8	ST239-III [3A]	*agr*_I	4	1	3	12	VISA, DS	Vanc 15 d	[4], [15]
***Pair 5***											
JKD6052	Australia	8	ST239-III [3A]	*agr*_I	1	0.25	1	2	VSSA, DS		[4], [15]
JKD6051		8	ST239-III [3A]	*agr*_I	4	0.5	4	>32	VISA, DS	Vanc 32 d	[4], [15]
**Additional Isolates**											
BPH0191	Australia	8	ST239-III [3A]	*agr*_I	2	1	3	24	hVISA, DS		This study
PC3	USA	5	ST5 – II [2A]	*agr*_II	8	3	8	>32	VISA, DNS		[6]
Sweden 307	Sweden	5	ST5 – II [2A]	*agr*_II	8	1.5	6	32	VISA, DNS		M. Wootton
VISA 3759	Scotland	8	ST247 – I [1B]	*agr*_I	4	0.5	4	>32	VISA, DS		[62]
BPH0062	Sth. Africa	8	ST247 – I [1B]	*agr*_I	2	1	3	16	hVISA, DS		Jan Bell
BPH0065	Hong Kong	8	ST239-III [3A]	*agr*_I	8	3	16	>32	VISA, DNS		Jan Bell
BPH0073	Taiwan	8	ST239-III [3A]	*agr*_I	4	1	6	16	VISA, DS		Jan Bell
BPH0088	Japan	5	ST5 – II [2A]	*agr*_II	2	0.38	3	8	hVISA, DS		Jan Bell

**NB**. ^a^The number of days of *in vivo* vancomycin exposure between the first and last isolate in the pair. BMD, broth microdilution; Etest GRD, Etest for glycopeptide resistance detection; VSSA, vancomycin-susceptible *S. aureus*; VISA, vancomycin-intermediate *S. aureus*; hVISA, heterogenous-VISA; DNS, daptomycin non-susceptible; dapto, daptomycin; vanco, vancomycin; teico, teicoplanin.

### Genome Comparisons Highlight Mutations in *walKR* Associated with Reduced Vancomycin and Daptomycin Susceptibility in *S. aureus*

In an earlier study of the VSSA/VISA pair JKD6009/JKD6008 we compared partially assembled 454 GS20 sequences and found six nucleotide substitutions in JKD6008 [18]. We then showed that a mutation occurring in the sensor region of the *graS* gene partly explained the reduced vancomycin susceptibility of this strain. To now comprehensively address the question of mutations that contribute to VISA we used our recently completed JKD6008 reference genome [21] which we have shown is closely related to other Australasian ST239 strains (unpublished data), and is therefore an appropriate reference genome for analysis, and used our read-mapping technique to re-examine the genetic differences between JKD6008 and JKD6009 as well as comparing four other clinical VSSA/VISA pairs (Table 1, strain pairs 1, 2, 4 and 5). Using either SOLiD or Illumina technologies, high coverage short-read sequences were obtained for the clinical pairs with high mean fold coverage (JKD6009 [SOLiD] mean fold coverage 338.7x; other strains [Illumina] mean fold coverage 85.5 to 230.6x). The list of differences between JKD6009 and JKD6008 increased from six to 10, but each pair presented a limited list of mutations in the VISA strain compared to its VSSA parent (Table 2). The most interesting observation was the presence of a previously undetected SNP in the *walK* gene of JKD6008. Strikingly, three of the four other clinical pairs also had single mutations within the *walKR* locus (Table 2). In one pair (JKD6004/JKD6005), the only mutation identified was a single SNP in *walR* of VISA strain JKD6005. JKD6051 was the only VISA strain among the five sequenced pairs without a *walKR* mutation. Mutations of potential interest in this strain included a mutation in the regulator SarR (A68T), and a mutation in RpoB (H481Y).

**Table 2:** Results of whole genome sequence comparison of five pairs of VSSA and VISA.

Isolate Pair and Mutation no.	Mutation in VISA (Sa_JKD6008 coordinate)	Locus (Sa_JKD6008)	Gene	Function	Effect of Mutation
**Pair 1 - JKD6000/JKD6001**					
**1**	G to A (24673)	SAA6008_00018	*walR*	Response regulator, essential	A96T
**2**	G to A (1217720)	Intergenic			
**3**	T to A (2006385)	SAA6008_01867		Putative phage protein	Q22H
**4**	G to A (2142758)	SAA6008_02029		dUTPase	Silent
**5**	T to G (2142774)	SAA6008_02029		dUTPase	N2T
		SAA6008_02030		acetyltransferase	E136D
**6**	T to C (2150689)	Intergenic			
**7**	C to T (2151557)	Intergenic			
**Pair 2 - JKD6004/JKD6005**					
**1**	A to G (25010)	SAA6008_00018	*walR*	Response regulator, essential	K208R
**Pair 3 - JKD6009/JKD6008**					
**1**	G to A (25769)	SAA6008_00019	*walK*	Sensor kinase, essential	G223D
**2**	A to G (360905)	SAA6008_00313	*glpT*	Glycerol-3-phosphate transporter	Silent
**3^a^**	C to T (734466)	SAA6008_00676	*graS*		T136I
**4^a^**	G to A (976097)	SAA6008_00920	*addA*	ATP-dependent nuclease subunit A	Silent
**5^a^**	T to A (1721456)	SAA6008_01608	*tgt*	Queuine tRNA-ribosyltransferase	F365Y
**6**	C to T (2391832)	Intergenic			
**7^a^**	C to T (2470905)	SAA6008_02357		Sodium/bile acid symporter family protein	P128S.
**8^a^**	G to A (2754296)	SAA6008_02622		Pyridine nucleotide-disulphide reductase	G268D
**9^a^**	C to T (2846492)	Intergenic			
**10**	G to A (2892543)	SAA6008_02743		ABC transporter ATP binding protein	Silent
**Pair 4 - JKD6021/JKD6023**					
**1**	G to T (25903)	SAA6008_00019	*walK*	Sensor kinase, essential	V268F
**2**	G to A (57470)	SAA6008_00049		Cadmium-transporting ATPase	A163T
**3**	C to T (454602)	Intergenic			
**4**	C to A (637292)	Intergenic			
**5**	A to T (727814)	SAA6008_00669		Hypothetical Protein	Premature stop
**6**	T to C (916693)	Intergenic			
**7**	G to A (2022797)	Intergenic			
**8**	G to A (2403501)	SAA6008_02288	*rpsJ*	30S ribosomal protein S10	Silent
**Pair 5 - JKD6052/JKD6051**					
**1**	C to A (46858)	SAA6008_00039	*mecA*	PBP2a	A97S
**2**	C to T (600465)	SAA6008_00548	*rpoB*	DNA directed RNA polymerase beta subunit	H481Y
**3**	G to A (849972)	SAA6008_00779	*trxB*	Thioredoxin-disulfide reductase	A311T
**4**	T insertion (1015671)	SAA6008_00955		CHP	Premature stop
**5**	T to C (1062271)	SAA6008_01000	*menB*	Naphthoate synthase	Silent
**6**	C to T (2443041)	SAA6008_02331	*sarR*	Staphylococcal accessory regulator R	A68T
**7**	G to A (2892543)	SAA6008_02743		ABC transporter ATP binding protein	Silent

**NB**. ^a^These mutations were previously detected and described prior to genome closure and re-analysis. VSSA, vancomycin-susceptible *S. aureus*; VISA, vancomycin-intermediate *S. aureus*.

### Frequency and Role of *walKR* Mutations in Reduced Vancomycin and Daptomycin Susceptibility in *S. aureus*

Given the previous reports of diverse genetic pathways involved in VISA we were surprised to find a single locus that was mutated in four of our five pairs of sequenced strains. To extend this analysis the *walKR* locus was sequenced from 8 additional, unpaired global isolates of hVISA/VISA that were available for study (Table 3). This demonstrated that, in addition to the four VISA strains in the genome sequencing analysis, 6 of the 8 additional resistant strains also had a mutation in the *walKR* locus. The downstream genes *yycHIJ* are involved in repression of *walR* in *B. subtilis* [25], therefore we sequenced *yycHIJ* for the two strains where a mutation in *walKR* was not detected but no mutations were found (Table 3).

**Table 3:** Screening for *walKR* mutations in unique (non-paired) strains.

Isolate	Molecular Type	Phenotype	WalK	WalR	YycHIJ
BPH0191	ST239-III [3A]	hVISA, DS	del 469-470	ND	-
PC3	ST5 – II [2A]	VISA, DNS	A567D	ND	-
Sweden 307	ST5 – II [2A]	VISA, DNS	del 337-340	ND	-
VISA 3759	ST247 – I [1B]	VISA, DS	ND	ND	ND
BPH0062	ST247 – I [1B]	hVISA, DS	ND	ND	ND
BPH0065	ST239-III [3A]	VISA, DNS	T595I	ND	-
BPH0073	ST239-III [3A]	VISA, DS	N382S	ND	-
BPH0088	ST5 – II [2A]	hVISA, DS	L14F	ND	-

**Note:** DS, daptomycin susceptible; DNS, daptomycin non-susceptible; -, *yycHIJ* amplification and sequencing not performed in these strains.

**Figure 1:**
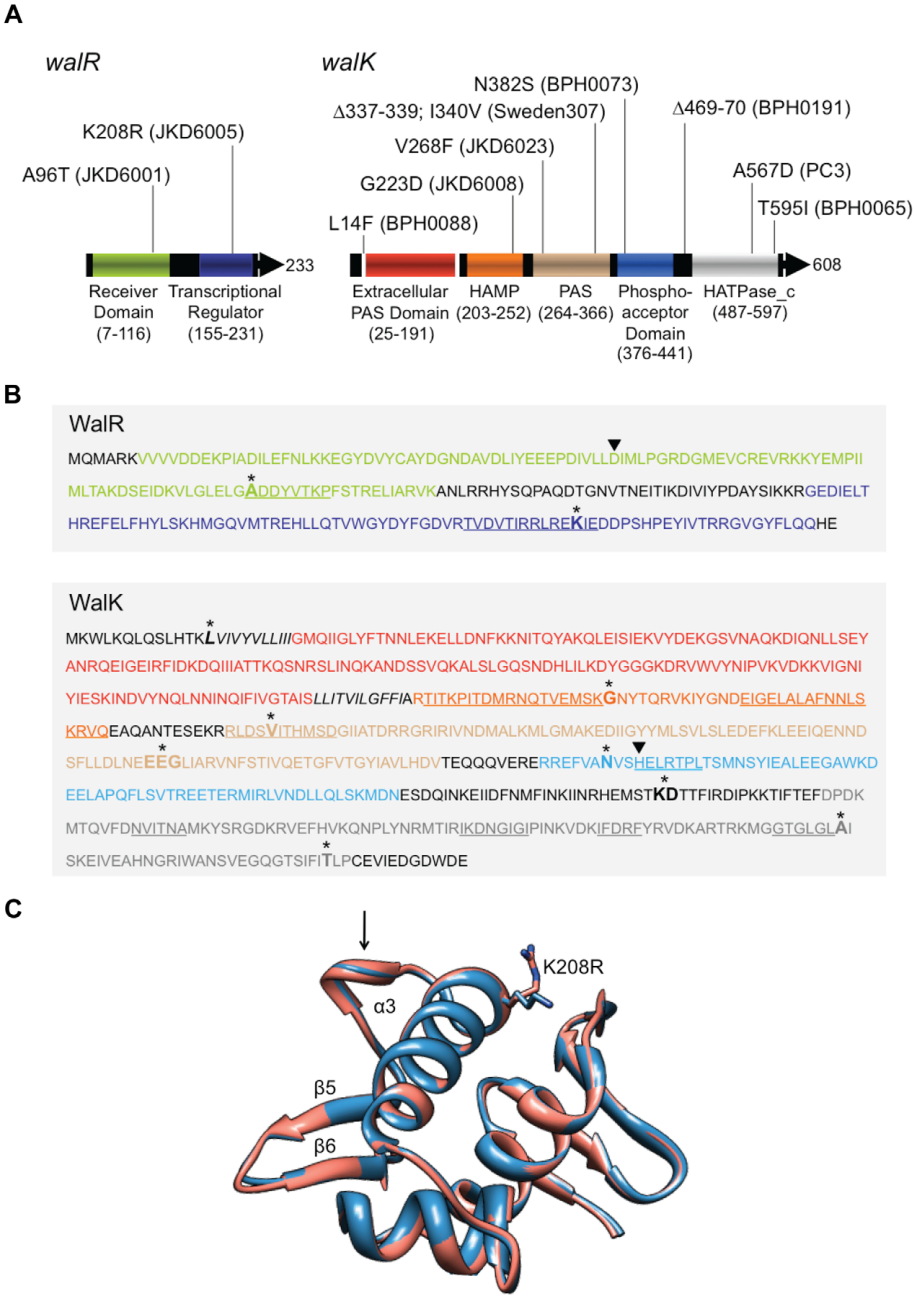
Analysis of location of *walKR* mutations detected in study isolates. (*A*) Schematic figure of the *walKR* operon showing protein domains and positions of identified mutations. Protein domains were defined according to Dubrac *et al* [25]. White regions of *walK* represent membrane spanning regions. (*B*) Amino acid sequences of the *walR* and *walK* genes. Domains are highlighted in color according to figure 1*A*. Identified mutations are indicated in bold type with an asterix. Membrane spanning regions of *walK* are italicized. The *walR* aspartic acid that is phosphorylated by *walK* and the *walK* histidine in the HisKA domain that is autophosphorylated are shown by arrows. Conserved regions are underlined and referred to in the text. (*C*) Ribbon diagram of the DNA binding domain of a WalR monomer from VISA strain JKD6005 (pink) modelled against the 1.87 Å structure of the WalR DNA-binding domain (PDB: 2ZXJ) from VSSA *Staphylococcus aureus* (blue). Shown is the position of the substituted amino acid (K->R) near the critical α3–β5 DNA binding loop (indicated by the arrow).

Analysis of the positions of the mutations within the *walKR* genes in this study indicates that they are not limited to a specific domain or region (Figure 1*A*). Indeed, the mutations occur across the spectrum of the domains that contribute to two-component regulator function. Each change therefore presumably exerts its effect via distinct mechanisms. For example the WalR A96T mutation occurs in a conserved region that is important for phosphorylation-mediated protein conformational changes [26] (Figure 1*B*), while the K208R mutation occurs in a highly conserved α3 DNA recognition helix region (Figure 1*B*). Recently, the crystal structure of DNA-binding domain of *S. aureus* WalR protein has been solved, and the interaction of the protein with target DNA examined [27]. Modelling the WalR DNA-binding domain with the K208R mutation from the VISA strain JKD6005 highlights its proximal location to the critical α3–β5 DNA binding loop (Figure 1*C*).

Within WalK, amino acid substitutions were detected in a range of functional domains (Figure 1), including the G223D mutation in JKD6008 at a highly conserved residue required for a sharp reverse turn between the α1 helix and the connector region of the HAMP domain [28], the 3-amino acid deletion Δ337–340 in BPH0191 in the β scaffold of the PAS domain, which is also known to be involved in its structural integrity [29], and the N382S mutation in WalK from BPH0073 in the phosphor-acceptor domain, only 3 amino acids from the conserved histidine that undergoes autophosphorylation and is essential for autokinase activity (Figure 1*B*).

### Generation and Whole Genome Sequencing of *walKR* Mutants

To measure the impact of these single nucleotide changes in *walKR*, bi-directional allelic replacement experiments were performed using two of the clinical pairs (Table 2, pairs 2 and 3). The *walK* mutation from VISA strain JKD6008 was introduced into the parent VSSA JKD6009, generating TPS3130 (Table 4). We have previously generated a GraS T136I mutation in JKD6009 (JKD6208) [18], therefore to measure the impact of the *walK/graS* double mutation, the *walK* mutation from VISA JKD6008 was also introduced into the previously produced *graS* mutant (JKD6208), generating TPS3128 (Table 4). The *walR* allele from the VISA strain JKD6005 was used to replace the *walR* allele in the VSSA parent JKD6004 generating TPS3190, and the *walR* allele from VSSA parent (JKD6004) was used to replace the *walR* allele from VISA strain (JKD6005) generating the vancomycin-susceptible strain TPS3124 (Table 4).

**Table 4:** Impact of mutations in WalKR on vancomycin and daptomycin susceptibility.

Isolate	Description	Standard vanco Etest (µg/mL)	Standard dapto Etest (µg/mL)	Macro Etest vanco 48hs (µg/mL)	Macro Etest teico 48hs (µg/mL)
**Mutant set 1**					
JKD6009	Parent VSSA	1.5	0.19	4	6
JKD6008	Clinical VISA	4	0.5	12	16
JKD6208	JKD6009, GraS T136I	2	0.25	6	8
TPS3130	JKD6009, WalK G223D	3	0.25	8	16
TPS3128	JKD6009, GraS T136I and WalK G223D	4	0.75	12	16
**Mutant set 2**					
JKD6004	Parent VSSA	1.5	0.5	4	6
JKD6005	Clinical VISA	4	2	8	12
TPS3124	JKD6005, WalR R208K (wildtype) from JKD6004	1.5	0.75	4	6
TPS3190	JKD6004, WalR K208R	4	2	8	12

**Note:** vanco, vancomycin; dapto, daptomycin; teico, teicoplanin; macro Etest uses a 2 McFarland inoculum.

We next sequenced the genomes of *S. aureus* mutants TPS3130 and TPS3190 to determine if unintended mutations had been introduced during the pKOR1-mediated allelic exchange process, particularly in other regulatory loci such as *agr*. Coverage of the reference strain JKD6008 was 99.7% and 96.9% for TPS3130 and TPS3190, respectively. For strain TPS3190, Ion Torrent sequencing confirmed the expected *walR* mutation at position 25010 and the presence of a single silent mutation in *walK* at position 26026, but no other changes. The unintended change at nucleotide 26026 was a PCR-induced error, introduced during cloning in pKOR1. The situation with strain TPS3130 was more complex. TPS3130, which is VSSA strain JKD6009 modified by replacing its *walK* gene with the allele from VISA strain JKD6008 (conferring the G223D amino acid change) had the predicted SNP at position 25769 (Figure S1) but also carried an additional four SNPs not present in JKD6009 (Table 5). Two of these four SNPs were the same as changes found in VISA strains JKD6008 and we propose that these might be compensatory mutations linked to the *walK* mutation. The probability of these changes occurring by chance at exactly these positions in JKD6008 and TPS3190 is small (*p*<2.3E13) and one can exclude the possibility of a strain mix-up, as the remaining two SNPs were specific to TPS3130 (Table 5). PCR and Sanger sequencing confirmed that the sequences at all five of these altered loci in JKD6009, JKD6008 and TPS3130 were correct.

**Table 5:** Summary of genomic differences between *S. aureus* TPS3130 versus JKD6009 and JKD6008.

No	Location (JKD6008)	Strain/SNP^a^			Locus_tag/annotation/comment
		JKD6000 (VSSA)	JKD6008 (VISA)	TPS3130 (VISA)	
1.	25769	GGT	GAT	GAT	SAA6008_00019. Sensor histidine kinase, WalK. Targeted allele swap in TPS3130, G223D.
2.	360905^b^	GCT	GCC	GCG	SAA6008_00313. Putative glycerol-3-phosphate transporter, GlpT. Silent mutation, same as JDK6008.
3.	517284^c^	A	A	*absent*	SAA6008_00478. PTS system trehalose-specific IIBC component, pseudogene. ‘A’ deletion in TPS3130 corrects frame-shift mutation found in JKD6009 and JKD6008.
4.	803235^c^	GAA	GAA	GGA	SAA6008_00741. Putative lipid kinase. Predicted amino acid change in TPS3130, E225G. Different to JKD6008 and JKD6009.
5.	2470905^b^	CCT	TCT	TCT	SAA6008_02357. Sodium/bile acid symporter family protein. Predicted amino acid change in TPS3130, P128S. Same as JKD6008.

a All mutations checked in all strains by PCR and Sanger sequencing.

b Indicates potential compensatory mutation accompanying the introduced *walK* mutation.

c Mutation only present in TPS3130.

**Table 6:** Laboratory strains, mutant strains, and plasmids used in study.

Strain or Plasmid	Features	Reference
**Laboratory Strains**		
RN4220 (*S. aureus*)	*S. aureus* strain capable of maintaining shuttle plasmids	[63]
*E. coli* DH5alpha		NEB
**Mutant Strains**		
JKD6208	JKD6009 with *graS* point mutation from VISA JKD6008, resulting in T136I mutation	[18]
TPS3128	JKD6208 with *walK* mutation from VISA JKD6008, resulting in G223D mutation (double mutant – *graS* and *walK*)	This study
TPS3130	VSSA JKD6009 with *walK* mutation from VISA JKD6008, resulting in G223D mutation	This study
TPS3124	VISA JKD6005 with wildtype *walR* allele from VSSA JKD6004	This study
TPS3190	VSSA JKD6004 with *walR* point mutation from VISA JKD6005, resulting in K208R mutation	This study
USA300 (FPR3757)	Clinical strain of ST8 clone USA300	[64]
**Plasmids**		
pKOR1	*E. coli* - *S. aureus* shuttle vector	[65]
pTPS6004	pKOR1 with *walR* allele from JKD6004, region for recombination generated with oligos 1901 and 1908	This study
pTPS6005	pKOR1 with *walR* allele from JKD6005, region for recombination generated with oligos 1901 and 1908	This study
pTPS6008	pKOR1 with *walK* allele from JKD6008, region for recombination generated with oligos 1901 and 1908	This study

### Impact of *walKR* Mutations on Antimicrobial Resistance

**Figure 2:**
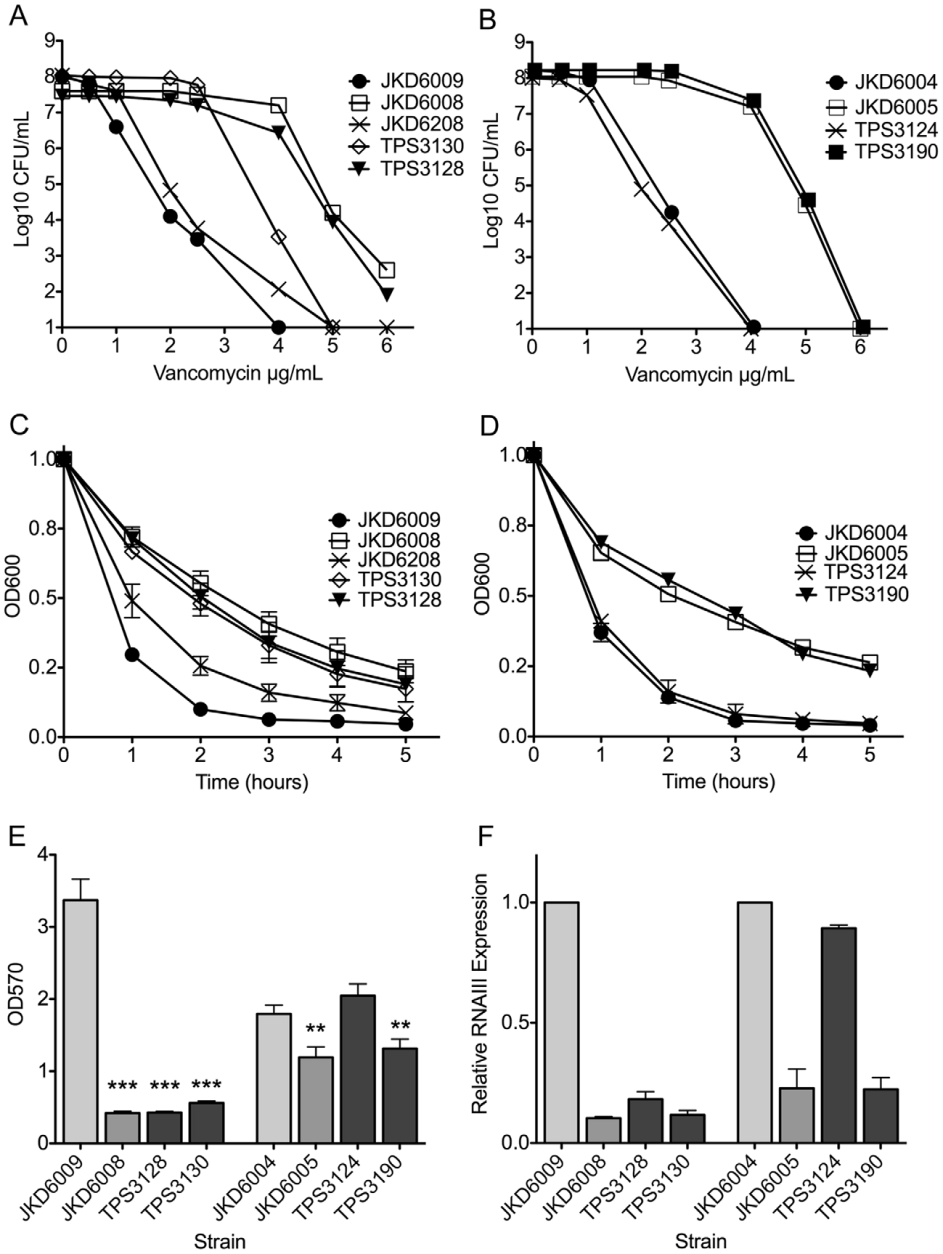
Analysis of antibiotic susceptibility, autolytic activity, biofilm formation and *agr* activity of clinical and mutant strains. (*A*) and (*B*) Vancomycin population analysis results demonstrate increased resistance in strains containing *walK* or *walR* mutations. (*C*) and (*D*) Autolytic activity for clinical and mutant strains demonstrates reduced autolytic activity in strains containing *walK* or *walR* mutations. All results are expressed as mean ± SEM for at least 3 biological replicates. (*E*) Biofilm analysis of clinical and mutant strains using a 96-well plate biofilm assay. The presence of *walK* or *walR* mutations significantly reduces biofilm formation compared to parental strains, mimicking the impact of the VISA phenotype on biofilm formation. All results are expressed as mean ± SEM for at least 3 biological replicates. Statistical analysis was performed comparing VISA and mutant strains to the parental strains JKD6009 or JKD6004 (** p<0.01; *** p<0.001). (*F*) Analysis of *agr* activity of clinical and mutant strains by real time quantitative PCR of the effector molecule RNAIII. Results are presented relative to the parental strain JKD6009 or JKD6004 which has been normalized to 1. All results are expressed as mean ± SEM for at least 3 biological replicates.

Vancomycin susceptibility significantly decreased when either the *walK* allele from JKD6008 (VISA) or the *walR* allele from JKD6005 (VISA) was swapped into its respective VSSA parent (Table 4 and Figure 2*A and B*). While the single G223D mutation in WalK converted the VSSA parent strain JKD6009 to VISA, the combination of the G223D mutation in WalK and the T136I mutation in GraS was required to convert JKD6009 to the full intermediate resistance of JKD6008. In comparison, the single K208R mutation in WalR from VISA strain JKD6005 was sufficient to convert JKD6004 (VSSA parent) to intermediate vancomycin resistance.

The *in vivo* evolution of VISA occurred without daptomycin exposure, however an increase in daptomycin MIC was detected in the majority of VISA strains in this study. Examination of daptomycin susceptibility in the mutants (Table 4) showed that these mutations in *walKR* promoted daptomycin co-resistance. The single WalR K208R substitution increased the daptomycin MIC of the parental VSSA strain JKD6004 from 0.5 µg per ml to 2 µg per ml generating a daptomycin non-susceptible strain (TPS3190), while the double *walK*/*graS* mutant (TPS3128) had a distinct increase in daptomycin MIC compared to the parental strain, although it remained within the defined susceptible range.

### The Impact of *walKR* Mutations on Other Recognized VISA Phenotypes

**Figure 3:**
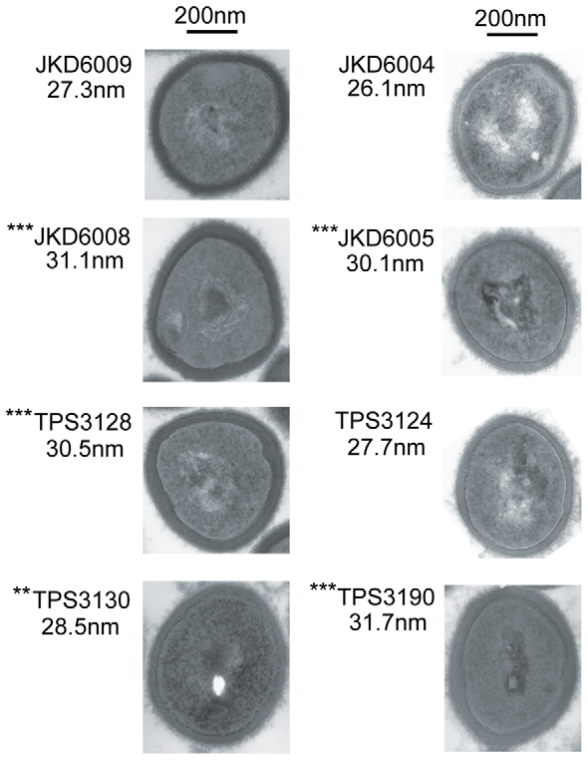
Cell wall thickness of clinical and *walKR* mutant strains. Representative electron microscopic images of parental and mutant *S. aureus* cells. The mean cell wall thickness for each strain is included, based on at least 100 measurements of the cell wall. Statistical analysis of cell wall thickness was performed compared to the parent strains JKD6009 or JKD6004. Note, *** p<0.001; ** p<0.01.

We next measured autolytic activity and cell wall thickness in the *walKR* allele-swapped strains compared to their VSSA and VISA parents. The impacts of the single substitutions in either WalR or WalK were dramatic, with significant reductions in autolytic activity and increases in cell wall thickness linked to the introduction of the *walR* or *walk* allele from the VISA strain into the VSSA parent (Figure 2*C* and 3). Conversely, the phenotypes were reversed when the VSSA *walR* allele from JKD6004 replaced *walR* in VISA strain JKD6005 (Figure 2*C* and 3).

**Figure 4:**
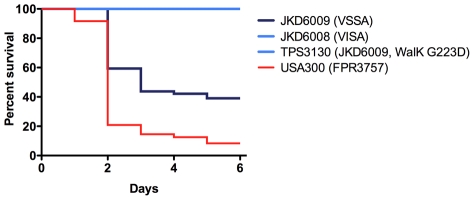
*Galleria mellonella* virulence analysis of clinical and *walK* mutant strains. Virulence analysis using the *Galleria mellonella* model demonstrating percent survival over 6 days using a Kaplan Meier plot. The parent VSSA strain JKD6009, the clinically derived VISA JKD6008, and the laboratory derived mutant TPS3130 are included. Included also for comparison is the virulent community clone of methicillin-resistant *S. aureus*, USA300. The difference in survival between JKD6009 and JKD6008 or TPS3130 was statistically significant (p<0.0001).

In our previous studies using these VSSA and VISA clinical pairs we had detected significant changes in a number of key staphylococcal virulence mechanisms in VISA [4]. Most strikingly, the VISA strains had marked reduction in *agr* activation despite the absence of mutations in the *agr* locus. We measured expression of RNAIII, the effector molecule of the *agr* locus, in the *walKR* mutants during exponential growth phase (Figure 2*F*) and showed that the mutations in either *walK* or *walR* had dramatic effects on *agr* expression with minimal RNAIII expression in VISA strains or the mutants harbouring the VISA *walKR* alleles compared to the VSSA parents. Additionally, it has previously been demonstrated using the *Galleria mellonella* model that clinical VISA has reduced virulence compared to parent strains [30], however the genetic basis for this has not been defined. Using this model the clinical VISA strain JKD6008, and the *walK* mutant TPS3130 demonstrated significantly reduced virulence compared to the parent strain JKD6009 (Figure 4), indicating that WalKR also plays an important role in control of virulence. The parent VSSA strain JKD6004 was relatively avirulent in this model, therefore significant changes in the clinical VISA (JKD6005) and the mutant strain (TPS3190) were not observed.

While biofilm formation is reduced in some VISA strains, the mechanisms underlying this have been unclear. We found that mutations in *walK* and *walR* led to significant reduction in bioflm formation that mimicked the changes seen in the clinical VISA strains (Figure 2*E*). There was no significant difference in growth rates of mutant and clinical strains to explain these findings.

### Transcriptional Effects of WalK and WalR Mutations, and Implications for WalKR Control of Central Metabolism

**Figure 5:**
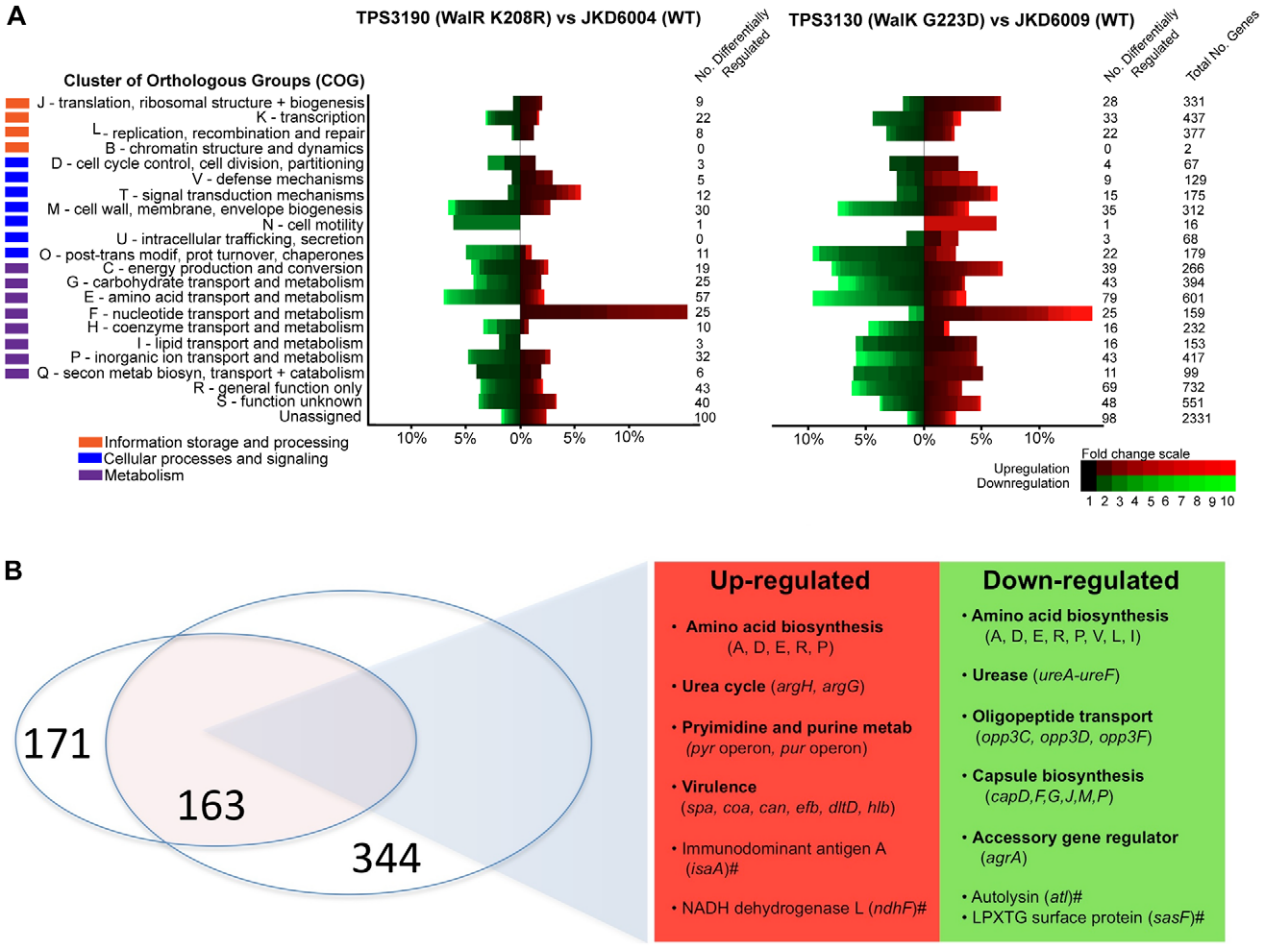
Microarray transcriptional analysis of defined WalK (G223D) and WalR (K208R) mutants compared to parental strains. (*A*) Results of microarray transcriptional analysis of TPS3130 (WalK G223D) and TPS3190 (WalR K208R) compared to respective parental strains. Up-regulated genes (in red) are differentially up-regulated in the mutants compared to the parent strains, and the down-regulated genes (in green) are differentially down-regulated in the mutant strains compared to parent strains. The heat map analysis highlights the proportion of each cluster of orthologous groups (COG) functional group [61] that is differentially regulated in the array analysis. This clearly demonstrates significant global transcriptional changes in the WalK and WalR mutant strains affecting genes from all COG groups, and providing a similar pattern of transcriptional response. (*B*) Schematic representation of the genes commonly transcribed in the two microarray experiments. A total of 163 genes were commonly transcribed, and the major up-regulated and down-regulated genes are highlighted. Note, # denotes loci which have been previously shown or are predicted to have upstream WalR binding domains [31], [32]; A, alanine; D, aspartate; E, glutamate; R, arginine; P, proline; V, valine; L, leucine; I, isoleucine.

We next used microarray analysis to explore the global regulatory effects of the *walK* (TPS3130) and *walR* (TPS3190) mutants compared to the parent strains (Figure 5*A, B*; Table S1). Any gene that demonstrated a fold change ≥1.5 accompanied by an adjusted *p*-value of <0.05 was included in the comparisons, and this included 507 genes in the TPS3130 vs JKD6009 analysis and 334 genes in the TPS3190 vs JKD6004 analysis. An analysis of genes that were significantly differentially regulated in the two experiments, in the same direction, revealed 90 genes that were down-regulated, and 73 genes that were up-regulated in WalKR mutants compared to parent strains (Table S1 and Figure 5).

Three genes in *S. aureus* have been previously shown to have upstream *walR* biding sites (orthologs in JKD6008 are SAA6008_02607, *isaA*; SAA6008_02335, *ssaA*_1; SAA6008_00250, *lytM*) [31], and six other genes involved in cell wall metabolism and virulence have predicted upstream *walR* binding sequences, and have been shown to have increased expression upon *walKR* induction in a mutant with inducible *walKR* activity [32]. Unexpectedly, only two of these genes were differentially regulated in both experiments. SAA6008_02607 (*isaA*) was up-regulated in both experiments, 2.3-fold in TPS3190 compared to parent and 6.2-fold in TPS3130 compared to parent, while SAA6008_1007 (*atl*) was down-regulated in both experiments, 1.8-fold (Table S1). Among 24 genes with predicted upstream WalR binding sequences [31], only two were differentially regulated in both our microarray experiments. These genes included SA6008_00455 (*ndhF*) encoding an NADH dehydrogenase subunit, which was up-regulated 2.2 to 2.4-fold, and SAA6008_02705 (*sasF*) encoding an LPXTG cell wall surface anchor protein, which was down-regulated 2.8 to 3.7-fold.

While WalKR has been identified as a master regulator of cell wall metabolism in *S. aureus* [25], the transcriptional profile of the two mutant strains in this analysis painted a different picture of WalKR control within the cell. The most dramatic and consistent transcriptional changes were found in genes involved in central metabolism. Using the Kyoto Encyclopedia of Genes and Genomes (KEGG) pathway analysis (www.genome.jp) [33], the major pathways where differential transcription occurred in both mutants were genes whose products are involved in amino acid metabolism (in particular alanine, aspartate, glutamate metabolism [up-regulation of *ald1*, *argG*, *argH*; down-regulation of *gltB*, *gltD*, SA6008_02661]; arginine and proline metabolism [up-regulation of *agrG*, *agrH*, and down-regulation of *aldA*, *ureA*, *ureB*, *ureC*]; valine, leucine and isoleucine metabolism [down-regulation of *ilvB*, *ilvC*, *ilvD*]), in addition to significant up-regulation of genes whose products are involved in purine and pyrimidine metabolism (*purF, purN, purM, purH, pyrC, pyrB, pyrAA, pyrR, pdp, tdk*). In contrast, only five out of 27 genes that have been consistently grouped as part of the staphylococcal “cell wall stimulon” (*sbtB*, *vraS*, *sgtB*, SA6008_01906, and SA6008_02595) [34]–[38] were differentially regulated in the two experiments. All five of these genes were down-regulated (1.6 to 3.6-fold) in the *walKR* mutant compared to the parent strain.

The veracity of the microarray data was checked by quantitative RT-PCR analysis of six genes from different pathways, including *argH*, *purF*, *pyrA*, *ureA*, *atl*, and *gltB*. The qRT-PCR data was consistent with the microarray results (Figure S2).

## Discussion

Methicillin-resistance in *S. aureus* remains a significant public health issue that is compounded by the evolution of strains with low-level resistance to last-line agents such as vancomycin and daptomycin. While the first VISA strain was reported in 1997 [5], our understanding of the genetic determinants of VISA remains incomplete. To address this issue we took a comparative genomics approach that included whole genome sequencing of 10 clinical *S. aureus* isolates, and showed that mutations within the essential *S. aureus* regulatory locus *walKR* are important mediators of intermediate vancomycin resistance. The contribution of both *walK* and *walR* mutations to vancomycin resistance was experimentally confirmed using bi-directional allelic exchange experiments. By swapping *walK* and *walR* alleles between VSSA and VISA strains we could make susceptible strains resistant and then revert resistant strains to susceptible. In addition, consistently observed features of VISA strains worldwide include cell wall thickening and reduced autolytic activity [6]-[8], [14], [15], and analysis of our *walKR* mutants showed the key role played by this locus in VISA, as single nucleotide changes in either *walK or walR* led to strains exhibiting these well-described VISA phenotypes.

Daptomycin is another cell wall active antibiotic with a different mode-of-action to vancomycin but there is increasing data demonstrating a link between VISA and daptomycin non-susceptibility in *S. aureus* [10], [19]. The clinical pairs of VSSA and VISA used in this study were selected from patients that had been treated with vancomycin, and not daptomycin. The vancomycin induced *walKR* mutations led to an increase in daptomycin MIC in clinical *S. aureus*, in some cases generating full daptomycin non-susceptibility. While *walK* mutations have been previously associated with daptomycin non-susceptibility in association with *mprF* mutations [20], no genetic manipulation of the *walKR* locus has been performed to confirm the contribution of these mutations to daptomycin resistance. Here we have shown for the first time the contribution of mutations in *walK* or *walR* (without *mprF* mutations) to reduced daptomycin susceptibility. Interestingly, the combination of the *graS* (T136I) and *walK* (G223D) mutation in JKD6008 was associated with a higher daptomycin MIC than either mutation alone (Table 4), suggesting an additive effect of these two mutations to increase both vancomycin and daptomycin resistance.

WalKR is highly conserved and specific to low GC gram-positive pathogens [25], and is the only known essential two component regulatory system in *S. aureus* [39], [40]. It has been best studied in *B. subtilis*, *S. aureus* and *S. pneumoniae* where cell wall metabolism genes dominate the predicted regulon of WalR [25]. A high frequency of *walKR* mutations was detected in this study, with mutations in eight of the 10 VISA, and two of the three hVISA strains tested, suggesting that mutations within this locus are a previously under-appreciated mediator of VISA. Our findings are supported by recent data from Shoji *et al* who found *walK* mutations were common in an international collection of VISA strains, although no *walR* mutations were reported [41]. While much attention has been focussed on the role of *vraRS* and *graRS* mutations in VISA [12], [13], [18], [41] none of the five sequenced strains in our study contained a *vraRS* mutation, and only one strain (JKD6008) contained a previously recognized *graS* mutation [18], suggesting these regulators are not the dominant mediators of VISA. It is unclear if there is a clone-specific pathway to VISA, such that we may have uncovered a frequent pattern of *walKR* mutation because we sequenced ST239 strains of *S. aureus*, the dominant hospital clone from our region. We attempted to address this potential bias by including a global collection of VISA isolates that were available to us for testing, and included non-ST239 strains. It is notable then that all three ST5 VISA strains tested also contained a *walK* mutation, however the ST247 strains and one of the ST239 strains did not contain *walKR* mutations, nor mutations in the downstream negative regulators of *walKR*, *yycHIJ*. Mutations of potential interest in the strain JKD6051 that is the only fully sequenced strain not to contain a *walKR* mutation included a mutation in the regulator *sarR* (A68T), and a mutation in *rpoB* (H481Y). There has been recent interest in the role of *rpoB* mutations in VISA, with the demonstration that an A621E amino acid change in RpoB leads to the hVISA phenotype [17], and more recently a link demonstrated between rifampicin resistance in *S. aureus* (due to *rpoB* mutations) and hVISA [42]. Therefore it is possible that the *rpoB* mutation detected in JKD6051 contributed to the VISA phenotype, however this was not specifically tested in this study.

Whole genome analysis of the allelic exchange VISA mutant TPS3190 using Ion Torrent sequencing confirmed the absence of any significant additional mutations in this laboratory derived strain, in particular an absence of *agr* mutations that might otherwise explain some of the described phenotypes. The sequencing of TPS3130 revealed a more complex story, with the detection of four additional SNPs, two of which were also present in the clinical VISA strain JKD6008 (Table 5). WalKR is thought to control cell-wall metabolism through sensing the lipid-II intermediate during peptidoglycan biosynthesis and regulating autolysin gene expression in this organism [25], however the basis for its essentiality is not well understood but appears to be at least partly related to WalKR control of cell wall metabolism [40]. It is therefore likely that *S. aureus* is very sensitive to changes in *walKR* because of its essential nature. We propose that the modification of *walK* in JKD6008 (and TPS3130) has been accompanied by at least two compensatory mutations. Both of these SNPs have occurred in genes linked to substrate transport. A deeper understanding of the role of WalKR will be required before we can say how the changes we observed in JKD6008 and TPS3130 might compensate for a mutation in *walK*. The two additional TPS3130-specific changes are equally intriguing and may also be compensatory, with a nucleotide deletion that corrects a frame-shift mutation in a gene encoding a component of the trehalose group translocation phosphate transporter and a predicted amino acid change in a hypothetical gene with a putative lipid kinase domain (Table 5).

Two key questions arising are how do mutations in *walKR* influence function of the regulator, and then cause VISA. Mwangi *et al* reported a mutation in the downstream negative regulator of WalKR, *yycH* that was associated with an increase in vancomycin resistance in a series of clinical isolates. It was predicted that this mutation would increase WalKR activity by reducing negative feedback to the regulator [11]. In another study, an IS256 insertion into the *walKR* promoter region was associated with a change in vancomycin resistance, and the authors predicted that a hybrid, overactive promoter was responsible for increased *walKR* expression and resistance [43]. In the present study, however, we found a range of SNPs across all the functional domains of WalKR in VISA strains, leading us to propose that decreased WalKR performance is probably the more likely consequence of these changes and that the VISA phenotype is initiated by a reduction in WalKR activity. WalKR has also been shown to positively regulate genes encoding enzymes with cell wall lytic activity. The reduction in autolytic activity and attenuated biofilm formation of the *walKR* mutants in this study also suggest that the mutations are restricting WalKR function, as these phenotypes have been previously demonstrated to occur in a conditional mutant with reduced WalKR activity [32]. We have also recently described a similar phenomenon where a point mutation in another essential *S. aureus* gene (*relA*) contributed to a reduction in function of the enzyme, but not complete absence of activity [44].

To further investigate the contribution of the *walK* and *walR* mutations to resistance we performed a global transcriptional profile of the mutant strains compared to parent strains (Figure 5). We were surprised to find a relative paucity of cell wall metabolism genes represented in the transcription expression data. We intentionally used a low fold cut off of 1.5 for inclusion of differentially expressed genes in the analysis, and despite this we only found four genes that were differentially expressed in both experiments that have been previously predicted to be part of the WalR regulon [31], [32]. While down-regulation of major autolysin expression (*atl*) was detected (1.8-fold in both mutants) and this could potentially explain the changes in autolytic activity detected in WalK and WalR mutants (Figure 2), the dominant theme of the transcriptional profiles was a change in transcription of genes encoding products involved in selected aspects of central metabolism. For example, pathway analysis based on our microarray data suggests WalKR interacts directly or indirectly with several components of the urea cycle. So while cellular metabolism in the mutants has been shifted away from anabolism of branched chain amino acids (valine, leucine and isoleucine), metabolites within the urea cycle, including glutamine, arginine and aspartate, have gained more prominence, with up-regulation of genes encoding enzymes that could increase yields of substrates that feed purine and pyrimidine biosynthesis. An increase in pyrimidine metabolism would tie in neatly with the observed increased cell wall thickness and decreased capsular polysaccharide in the *walKR* mutant strains. Two distinct pathways are used by *S. aureus* to synthesise UDP *N*-acetylglucosamine (UDP-GlcNAc), a key intermediate for both peptidoglycan and capsular polysaccharide biosynthesis. Pools of UDP-GlcNAc for peptidoglycan biosynthesis are produced by glycolysis and pyrimidine metabolism while UDP-GlcNAc for capsule biosynthesis is linked to gluconeogenesis and aminosugar metabolism [45]. Therefore an increase in pyrimidine metabolism might be predicted to increase pools of UDP-GlcNAc available for peptidoglycan synthesis, with a concomitant decrease in the availability of the same metabolite for capsule production.

Finally, this study has demonstrated a link between *walKR* mutations and a number of VISA associated phenotypes. While biofilm formation is reduced in some VISA strains, including those previously characterized strains used in this study [15], the mechanisms underlying this have been unclear. We found that mutations in *walK* and *walR* led to significant reduction in biofilm formation that mimicked the changes seen in the VISA strains (Figure 2*E*). Reduced *walKR* activity in a conditional mutant was previously associated with reduced biofilm production [32], and is consistent with the changes in biofilm formation seen with *S. aureus* mutants deficient in autolysis [46], [47]. Extracellular DNA (eDNA) that is released by autolysis is an important initial step in *S. aureus* biofilm formation [48]. We propose that there may be an absence of eDNA in VISA and that this may explain the biofilm defect observed in VISA strains with *walKR* mutations. The *agr* locus is a quorum sensing toxin regulatory system that is critical to *S. aureus* virulence [49]. Using real time RNAIII PCR and microarray transcriptional analysis we have demonstrated that mutation of *walKR* is associated with reduced *agr* activation during exponential growth, however it is unclear if this is a direct or indirect relationship. Little is known about the role WalKR plays in *S. aureus* virulence. While virulence studies have been performed using streptococcal species, the impact of *walK* mutants in these experiments has been conflicting [50], [51]. Using the invertebrate model *Galleria mellonella* a significant attenuation of virulence of the *walK* mutant strain TPS3130 was demonstrated (Figure 4). However, because there was no difference in the virulence of the clinical pair JKD6004/JKD6005, no impact of the *walR* mutation in the parental strain was discernable.

In conclusion, this study highlights the potential for single nucleotide changes in *S. aureus* to dramatically affect bacterial behaviour and antimicrobial resistance. Complete bacterial genome sequencing of carefully selected strains has revealed mutations in *walKR* as a common mechanism for *in vivo* evolution of multi-drug resistance in this pathogen, leading to daptomycin cross-resistance, and impacting virulence mechanisms of the organism. Additionally, transcription profiling of strains with single base substitutions in *walK* or *walR* indicate that this regulatory locus not only controls autolysis, but more generally impacts metabolic activities within the cell. Efforts to design therapeutic strategies based on inhibiting *walKR* should be aware of the potential impact on the organism of inducing mutations in this locus.

## Materials and Methods

### Strains and Growth Conditions

Bacterial strains and a description of their antibiotic susceptibility, geographical origin, and plasmids used in the study are listed in Tables 1 and 6. Staphylococcal strains were stored in glycerol broth at −80°C and subcultured twice onto Horse Blood Agar (Oxoid) before being used for any experiment. Unless otherwise indicated all *S. aureus* isolates were grown in BHIB (Oxoid), and *E. coli* grown in LB broth (Oxoid). When required media was supplemented with the following antibiotics at the indicated concentrations: for *E. coli*, ampicillin 100 µg/ml; for *S. aureus* RN4220, chloramphenicol 10 µg/ml; for *S. aureus* clinical isolates, chloramphenicol 25 µg/ml.

### Antibiotic Susceptibility and Molecular Typing

Vancomycin MICs were determined by microbroth MIC according to CLSI criteria [52], and VISA defined as a strain with a vancomycin broth MIC of 4–8 µg per ml [52]. Detection of heterogenous-VISA (hVISA) was performed by Etest GRD according to recommendations of the manufacturer, while clinical and mutant strain pairs were tested by Macro method Etest using a 2 McFarland inoculum [3]. Daptomycin MICs were performed by Etest (AB Biodisk), according to manufacturer’s instructions, and isolates were defined as daptomycin non-susceptible according to CLSI criteria (daptomycin MIC >1 µg per ml) [52]. Clinical isolates underwent molecular characterisation using the DNA microarray StaphyType96 (CLONDIAG, Jena, Germany). DNA extraction was performed using the DNeasy Tissue Kit (Qiagen, Hilden, Germany), and the microarray and data analysis were performed as previously described [53]. The DNA microarray assigns strains to clonal complexes (CC) using reference strains previously defined by multilocus sequence typing (MLST) and *spa* typing. Pulsed-field gel electrophoresis using *Sma*I enzyme restriction was performed using the CHEF DR III system (BioRad, Berkeley, California) to confirm clonal group if necessary [54].

### DNA Methods, Molecular Techniques and Construction of Mutants

Standard procedures were used for DNA manipulation, molecular techniques, PCR, sequencing and plasmid extraction. To generate *walK* and *walR* mutants, allelic replacement experiments were performed using the vector pKOR1, as described previously [18]. The locus containing the *walKR* mutation was amplified from JKD6008, JKD6004, and JKD6005 for exchange into the respective parental strains using primers 1901 and 1908 (Table S2). The amplified product was cloned into the *attB* sites of pKOR1 and then transformed into *E. coli* DH5alpha. After transformation into *E. coli*, pKOR1 with the integrated *walKR* locus was extracted and sequenced to confirm the correct sequence, prior to performing allelic exchange in RN4220 intermediate and the clinical *S. aureus* strain. To confirm that no other significant mutations were introduced during the homologous recombination, the whole *walKR* locus was sequenced from the mutant strains using oligonucleotides covering the whole replaced sequence and its flanking ends (Table S2).

### High Throughput DNA Sequencing

Genome sequences for four ST239 VSSA and VISA clinical pairs (VSSA: JKD6000, JKD6004, JKD6021 and JKD6052; VISA: JKD6001, JKD6005, JKD6023, and JKD6051) were obtained from an Illumina Genome Analyzer II using 36-cycle paired-end chemistry. SOLiDv2 26 bp mate-pair sequencing was also performed on the clinical VSSA strain JKD6009, for which previous 454 GS20 shotgun sequence data was available. Single-end genome sequencing of the two laboratory-induced mutants TPS3130 and TPS3190 was performed using Ion Torrent sequencing as described [55]. TPS3130 yielded 88.7 Mbp from four 314 chips and TPS3190 yielded 56.9 Mbp from two 314 chips.

### Comparative Genomics

A read mapping approach was used to compare the sequences from the four VSSA/VISA clinical pairs described above, the previously described clinical pair JKD6009/JKD6008 (NCBI Genbank accession numbers NZ_ABSA00000000 and CP002120) and the Ion Torrent sequences from TPS3130 and TPS3190. The reads from all genomes were aligned to the JKD6008 reference using SHRiMP 2.0 [56]. SNPs were identified using Nesoni v0.52, which uses the aligned reads of each genome to the reference to construct a tally of putative differences at each position, including substitutions, insertions, and deletions [www.bioinformatics.net.au]. This tally was input to a Bayesian model to decide whether a base (or deletion) could be called for the position, and if so, whether it differed from the reference. Each position is treated as possibly containing a mixture of bases. A particular base is called if the likelihood that it makes up more than 50% of the mixture exceeds a threshold, set to 0.99 by default. This likelihood is calculated by updating a prior distribution over all possible mixtures, in the form of a Dirichlet distribution, as bases are observed. A similar procedure is used to call the presence or absence of insertions between positions in the reference. Using the whole genome sequence of JKD6008 as a reference a global SNP analysis was performed, and allelic variability at any nucleotide position was tallied to generate a global SNP analysis for every genome compared to JKD6008. Protein modelling was performed using the crystal structure of the WalR DNA-binding domain of *S. aureus* (PDB structure: 2ZXJ) using ModBase [27], [57].

### Phenotypic Assays

Biofilm and autolytic assays were performed as described previously [15]. Cell wall thickness was measured by taking 100 readings of wall thickness per strain from multiple cells after preparation of electron microscopic images as described previously [15].

### *Galleria mellonella* Virulence Assay

This invertebrate *S. aureus* infection model was used to study the virulence of clinical and mutant strains as previously described [44]. Briefly, a HPLC syringe was used to inject 10 µL of bacterial suspension (approx 1.0×10^6^ CFU) into the last left proleg of each caterpillar. Bacterial colony counts were performed to confirm consistency of inoculum, and caterpillars monitored daily for 6 days. Multiple biological replicates were performed for each strain.

### Microarray Transcriptional Analysis

Microarray transcriptional analysis was performed with TIGR version 9 *S. aureus* arrays, as previously described [4]. For preparation of total RNA shaking flasks (50 mL BHI broth in 250 mL flasks) were inoculated with 500 µL overnight BHI broth culture and incubated on a 225rpm shaker at 37°C. Optical density was closely monitored, and one millilitre of sample was collected at exponential growth phase (optical density at 600 nm of 0.5) and 0.5 mL RNA stabilization reagent (RNA later, Qiagen) was added and mixed immediately. The mixture was allowed to stand in room temperature for 10 minutes before total RNA was extracted using the RNeasy micro kit (Qiagen). RNA extractions and hybridisations were performed on four different occasions, and the dye swapped with each biological replicate. The images were combined and quantified and then imported into BASE and analyzed using Bioconductor and Limma [58], [59]. The fold ratio of gene expression for the mutant strains TPS3130 and TPS3190 relative to the parental MRSA strain JKD6009 and JKD6004, respectively, were calculated. Using a modified t-test p values were calculated and adjusted for multiple testing using false discovery rate (FDR) correction. A ≥ 1.5-fold change with p<0.05 was considered significant.

### Quantitative RT-PCR

To investigate activity of the *agr* locus (RNAIII) and confirm the microarray transcriptional results qRT-PCR was performed for RNAIII, *argH*, *purF*, *pyrA*, *ureA*, *atl*, and *gltB*, using oligos in Table S2. RNA was prepared from exponential phase cultures as previously described [15]. Two on-column DNase I digestion steps were performed and cDNA synthesis using SuperScript II RNase H reverse transcriptase (Invitrogen) included a SuperScript II negative control to confirm the absence of genomic DNA. Relative expression was determined as previously described [15], and was normalised against *gyrB* as previously described [60]. Results were obtained from 3 biological replicates each performed in triplicate.

### Statistical Analysis

Statistical analyses of mutant strains were performed using the two-tailed Mann Whitney U test, with a p<0.05 set for statistical significance. Kaplan Meier plots of *G. mellonella* killing results were analyzed using the log rank test. All analyses were performed using Prism for Macintosh ver 5.0 (GraphPad Software Inc., CA, USA)

### Accession Numbers

For the sequenced clinical strains JKD6000, JKD6001, JKD6004, JKD6005, JKD6009, JKD6021, JKD6023, JKD6051, JKD6052 the reads have been deposited in the NCBI Sequence Read Archive under study accession number SRA027352. The Ion Torrent reads for the mutant strains TPS3130 and TPS3190 have been deposited in the NCBI Sequence Read Archive under study accession number SRA044879.2. Microarray data has been submitted to GEO with accession number GSE29157. Protein modelling was performed using the crystal structure of the WalR DNA-binding domain of *S. aureus* (PDB structure: 2ZXJ).

## Supporting Information

Figure S1 Results of Ion Torrent sequencing of mutant strain TPS3130. (*A*) Non-ambiguous read mapping of Ion Torrent sequences for TPS3130 against the whole genome sequence of reference strain JKD6008, demonstrating genome coverage and depth (repeat regions excluded). (*B*) Detailed analysis of the read coverage results for the *walKR* operon confirms the presence of the G to A mutation at position 25769 in the reference strain JKD6008 and the mutant TPS3130.
(TIF)

Figure S2 Quantitative real-time PCR confirmation of microarray results. Using qRT-PCR the fold ratio of gene expression for the mutant strain (TPS3130 or TPS3190) was compared to the parent strain (JKD6009 or JKD6004) for six genes (*argH*, *purF*, *pyrA*, *ureA*, *atl* and *gltB*). qRT-PCR results are presented as mean ± SEM for at least 3 biological replicates. Microarray expression results for the same genes also shown.
(TIF)

Table S1 List of differentially regulated genes common to the WalK (TPS3130) and WalR (TPS3190) mutants generated in this study compared to parental strains, based on global microarray transcriptional analysis.
(PDF)

Table S2 List of primers used in this study.
(DOCX)
