# Supplementary figures and images for: Evolution of Multidrug Resistance during Staphylococcus aureus Infection Involves Mutation of the Essential Two Component Regulator WalKR
Source: PLoS Pathog. 2011 Nov 10;7(11):e1002359. doi: 10.1371/journal.ppat.1002359 (PMC3213104; doi:10.1371/journal.ppat.1002359)

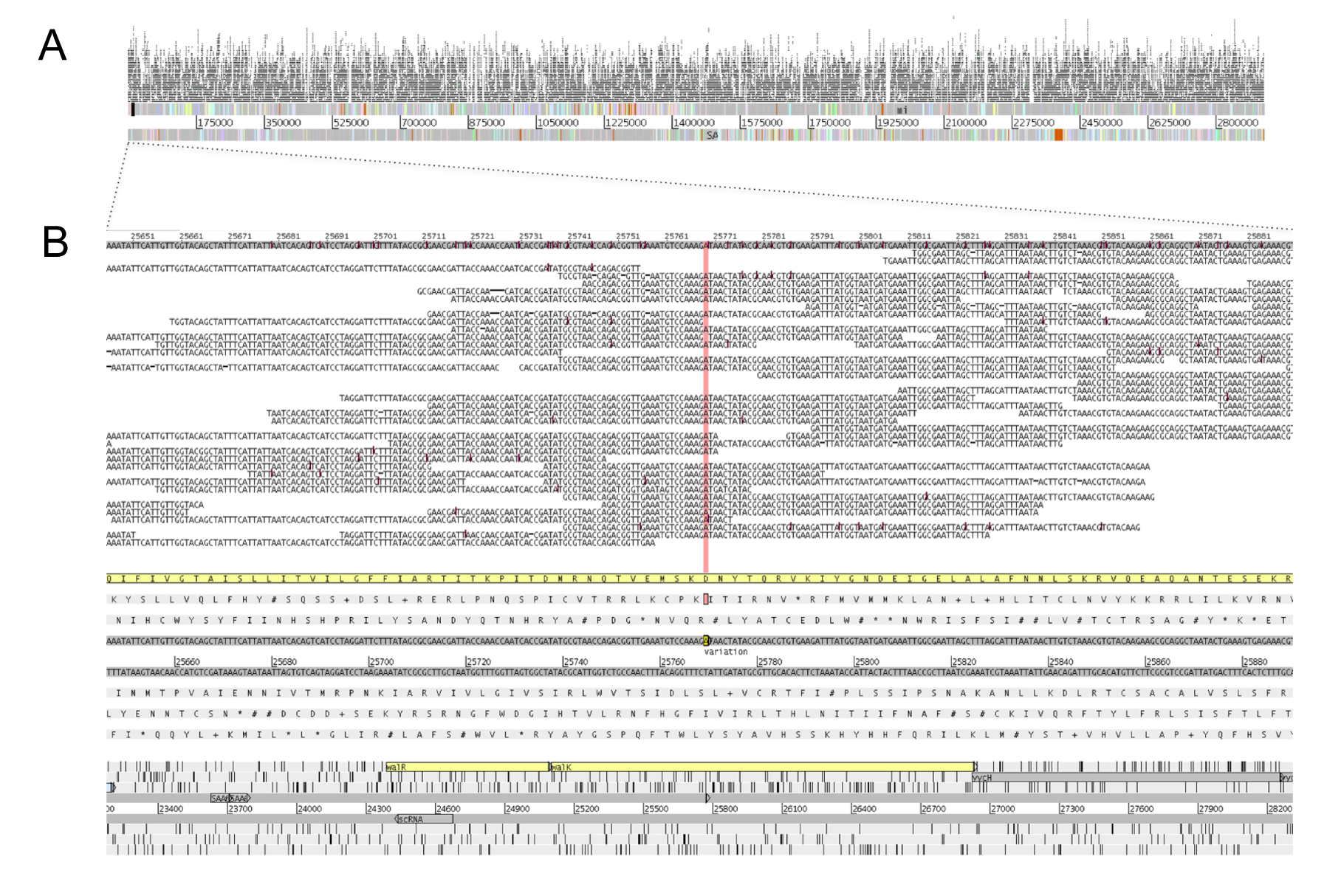

Supplement: Figure S1 — Results of Ion Torrent sequencing of mutant strain TPS3130. (A) Non-ambiguous read mapping of Ion Torrent sequences for TPS3130 against the whole genome sequence of reference strain JKD6008, demonstrating genome coverage and depth (repeat regions excluded). (B) Detailed analysis of the read coverage results for the walKR operon confirms the presence of the G to A mutation at position 25769 in the reference strain JKD6008 and the mutant TPS3130. (TIF) [file ppat.1002359.s001.tif]

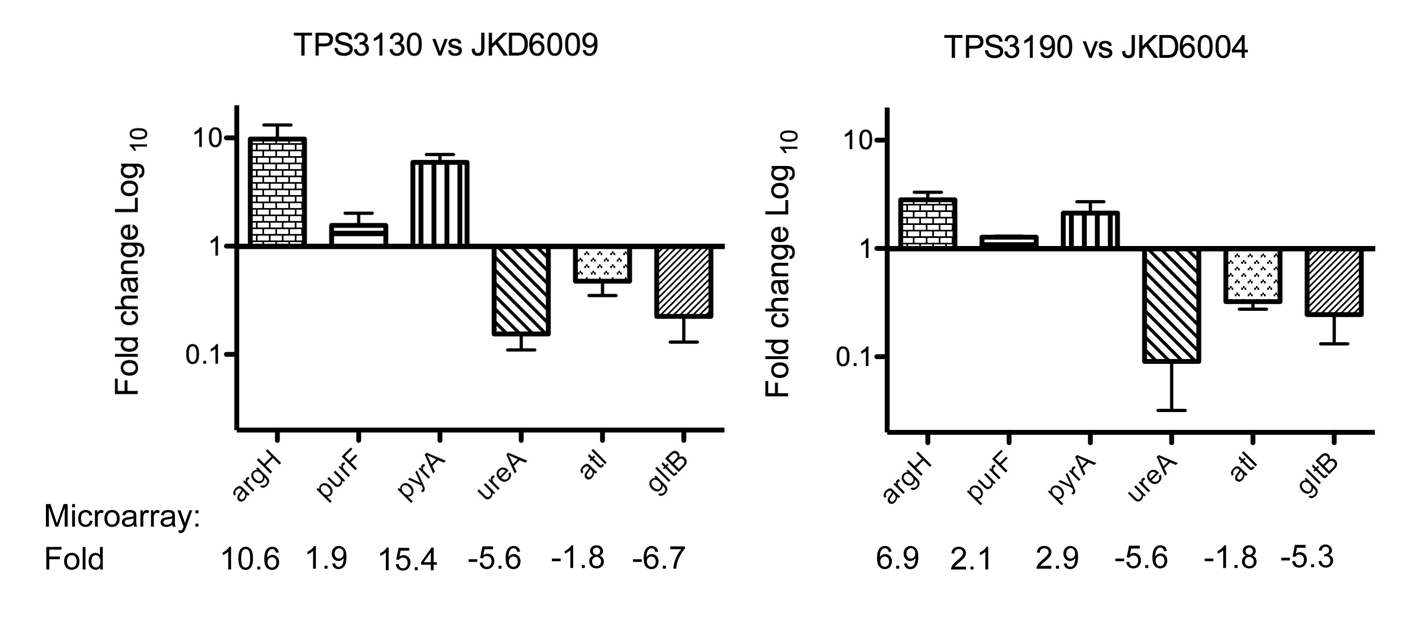

Supplement: Figure S2 — Quantitative real-time PCR confirmation of microarray results. Using qRT-PCR the fold ratio of gene expression for the mutant strain (TPS3130 or TPS3190) was compared to the parent strain (JKD6009 or JKD6004) for six genes (argH, purF, pyrA, ureA, atl and gltB). qRT-PCR results are presented as mean ± SEM for at least 3 biological replicates. Microarray expression results for the same genes also shown. (TIF) [file ppat.1002359.s002.tif]
