# Supplementary material for: Evolution of Multidrug Resistance during Staphylococcus aureus Infection Involves Mutation of the Essential Two Component Regulator WalKR
Source: PLoS Pathog. 2011 Nov 10;7(11):e1002359. doi: 10.1371/journal.ppat.1002359 (PMC3213104; doi:10.1371/journal.ppat.1002359)
Supplement: Table S1 — List of differentially regulated genes common to the WalK (TPS3130) and WalR (TPS3190) mutants generated in this study compared to parental strains, based on global microarray transcriptional analysis. (PDF) [file ppat.1002359.s003.pdf]

Table S1. Differentailly regulated genes common to both WalKR mutants compared to parent strains.

Saa_6008 Gene ID	TPS3190 vs JKD6004	Adjusted p value	TPS3130 vs JKD6009	Adjusted p value	COG	Gene	Description
**DOWNREGULATED**							
SAA6008_00036	0.66	0.007	0.52	0.037	COG3425I		conserved hypothetical protein (probable HMG-CoA synthase)
SAA6008_00083	0.53	0.044	0.36	0.012	COG1473R		peptidase, M20/M25/M40 family
SAA6008_00084	0.22	0.0081	0.13	0.0042	COG0477GEPR		major facilitator transporter
SAA6008_00105	0.62	0.0083	0.41	0.00089	COG1028IQR	*butA*	putative short chain dehydrogenase / acetoin reductase
SAA6008_00115	0.48	0.00058	0.6	0.00024			putative tetracycline resistance protein
SAA6008_00130	0.48	0.00099	0.24	0.0006	COG1086MG	*cap8D*	capsular polysaccharide biosynthesis protein CapD
SAA6008_00132	0.58	0.0016	0.11	0.0032	COG1898M	*cap8F*	capsular polysaccharide synthesis enzyme CapF
SAA6008_00133	0.5	0.0018	0.13	0.00034	COG0381M	*cap8G*	capsular polysaccharide biosynthesis protein CapG
SAA6008_00136	0.54	0.0023	0.19	0.0000039	COG0110R	*cap8J*	capsular polysaccharide synthesis enzyme CapJ
SAA6008_00139	0.64	0.0059	0.31	0.02	COG2148M	*cap8M*	capsular polysaccharide synthesis enzyme CapM
SAA6008_00142	0.63	0.00042	0.39	0.0094	COG0381M	*cap8P*	capsular polysaccharide biosynthesis protein CapP
SAA6008_00145	0.64	0.028	0.56	0.034	COG1012C	*aldA*	putative aldehyde dehydrogenase
SAA6008_00153	0.55	0.00054	0.42	0.00034	COG5609S		conserved hypothetical protein
SAA6008_00166	0.13	0.00011	0.1	0.00018	COG3961GHR	*ipdC*	thiamine pyrophosphate enzyme
SAA6008_00249	0.47	0.028	0.35	0.0084	COG3049M		penicillin V acylase
SAA6008_00302	0.56	0.046	0.27	0.0075	COG2110R		macro domain protein
SAA6008_00334	0.1	0.00066	0.13	0.0035	COG0646E	*metH*	bifunctional homocysteine S-methyltransferase
SAA6008_00336	0.25	0.0066	0.46	0.031	COG0626E		bifunctional cystathionine gamma-lyase/gamma-synthase
SAA6008_00421	0.46	0.0038	0.37	0.0013	COG0702MG		cobalt (Co2+) ABC superfamily ATP binding cassette transporter, membrane protein
SAA6008_00463	0.25	0.00055	0.04	0.0022	COG0031E	*cysM*	cysteine synthase
SAA6008_00464	0.45	0.0024	0.1	0.000089	COG0626E	*metB*	bifunctional cystathionine gamma-lyase
SAA6008_00475	0.19	0.0016	0.15	0.0033	COG0069E	*gltB*	glutamate synthase (NADPH), large subunit
SAA6008_00476	0.47	0.0016	0.51	0.0094	COG0493ER	*gltD*	glutamate synthase (NADPH) small subunit
SAA6008_00530	0.59	0.013	0.32	0.002	COG3869E		D-isomer specific 2-hydroxyacid dehydrogenase family protein
SAA6008_00660	0.64	0.00052	0.49	0.0027	COG1132V	*abcA*	ATP-binding cassette transporter A
SAA6008_00767	0.66	0.029	0.34	0.00064	COG1544J	*yfiA*	sigma 54 modulation protein/ribosomal protein S30EA
SAA6008_00835	0.5	0.00088	0.55	0.0037	COG1135P		DL-methionine transporter ATP-binding subunit
SAA6008_00836	0.35	0.00098	0.24	0.00013	COG2011P		binding-protein-dependent transport systems inner membrane component
SAA6008_00837	0.43	0.00061	0.2	0.012	COG1464P		ABC superfamily ATP binding cassette transporter, binding protein
SAA6008_00898	0.14	0.02	0.02	0.000058	COG1252C		pyridine nucleotide-disulphide oxidoreductase
SAA6008_00940	0.42	0.017	0.18	0.012	COG1173EP	*opp3C*	oligopeptide ABC superfamily ATP binding cassette transporter, membrane protein, Opp3C
SAA6008_00941	0.47	0.018	0.43	0.0052	COG0444EP	*opp3D*	oligopeptide ABC superfamily ATP binding cassette transporter, ABC protein, Opp3D
SAA6008_00942	0.65	0.036	0.31	0.012	COG1123R	*opp3F*	oligopeptide ABC superfamily ATP binding cassette transporter, ABC protein, Opp3F
SAA6008_01007	0.54	0.00073	0.55	0.0095	COG4193G	*atl*	bifunctional N-acetylmuramoyl-L-alanine amidase/endo-beta-N-acetylglucosaminidase, Atl
SAA6008_01127	0.25	0.012	0.17	0.00017			antibacterial protein (phenol soluble modulin)
SAA6008_01128	0.22	0.031	0.21	0.0047			antibacterial protein (phenol soluble modulin)
SAA6008_01131	0.55	0.0036	0.33	0.0048	COG4365S		conserved hypothetical protein
SAA6008_01343	0.61	0.0035	0.42	0.0045	COG1124EP	*nikE*	ABC-type dipeptide/oligopeptide/nickel transporter, ATPase component 2
SAA6008_01344	0.47	0.0048	0.27	0.00056	COG0444EP	*nikD*	ABC-type dipeptide/oligopeptide/nickel transporter, ATPase component 2
SAA6008_01379	0.65	0.035	0.34	0.0022	COG0567C	*kgd*	oxoglutarate dehydrogenase (succinyl-transferring) E1
SAA6008_01690	0.44	0.022	0.28	0.00034	COG1764O	*ohr*	organic hydroperoxide resistance protein
SAA6008_01692	0.51	0.00053	0.22	0.035	COG0111HE	*serA*	D-3-phosphoglycerate dehydrogenase / D-3-phosphoglycerate dehydrogenase
SAA6008_01819	0.57	0.0093	0.39	0.00056	COG0111HE		D-isomer specific 2-hydroxyacid dehydrogenase family protein
SAA6008_01828	0.63	0.0053	0.34	0.025	COG1134GM		ABC-type polysaccharide/polyol phosphate transport system ATPase component-like protein
SAA6008_01831	0.64	0.011	0.28	0.04	COG0744M	*sgtB*	monofunctional glycosyltransferase
SAA6008_01905	0.58	0.0016	0.45	0.0047	COG4585T	*vraS*	two-component sensor histidine kinase
SAA6008_01906	0.56	0.0048	0.52	0.0086	COG4758S		conserved hypothetical protein
SAA6008_02077	0.42	0.0091	0.39	0.013	COG3279KT	*agrA*	accessory gene regulator A
SAA6008_02091	0.2	0.03	0.42	0.000089	COG0129EG	*ilvD*	dihydroxy-acid dehydratase
SAA6008_02092	0.21	0.0072	0.18	0.0006	COG0028EH	*ilvB*	acetolactate synthase, large subunit, biosynthetic type
SAA6008_02093	0.39	0.02	0.2	0.0097	COG0440E		acetolactate synthase 1 regulatory subunit
SAA6008_02094	0.4	0.00088	0.31	0.015	COG0059EH	*ilvC*	ketol-acid reductoisomerase
SAA6008_02205	0.61	0.00088	0.66	0.049	COG2814G		drug resistance transporter EmrB/QacA subfamily protein
SAA6008_02218	0.24	0.023	0.18	0.035	COG2270R		major facilitator transporter
SAA6008_02240	0.36	0.0083	0.39	0.0011	COG0656R		oxidoreductase, aldo/keto reductase
SAA6008_02241	0.59	0.014	0.45	0.035	COG0789K		transcriptional regulator MerR family protein
SAA6008_02323	0.07	0.0047	0.23	0.023	COG4413E	*utp*	membrane-bound urea transporter
SAA6008_02324	0.18	0.001	0.18	0.00031	COG0831E	*ureA*	urease gamma subunit
SAA6008_02325	0.27	0.019	0.31	0.000082	COG0832E	*ureB*	urease beta subunit
SAA6008_02326	0.19	0.00082	0.26	0.000016	COG0804E	*ureC*	urease alpha subunit
SAA6008_02327	0.24	0.0029	0.31	0.000018	COG2371O	*ureE*	urease accessory protein
SAA6008_02328	0.25	0.001	0.25	0.000012	COG0830O	*ureF*	urease accessory protein
SAA6008_02330	0.35	0.0037	0.42	0.0065	COG0829O	*ureD*	urease accessory protein
SAA6008_02357	0.54	0.016	0.27	0.041	COG0385R		sodium/bile acid symporter family protein
SAA6008_02409	0.56	0.0015	0.49	0.013	COG0454KR		acetyltransferase, GNAT family
SAA6008_02410	0.6	0.014	0.46	0.000089	COG0492O		pyridine nucleotide-disulphide oxidoreductase
SAA6008_02488	0.63	0.0028	0.5	0.0071	COG1732M	*opuCC*	glycine betaine/L-proline ABC superfamily ATP binding cassette transporter, binding protein
SAA6008_02491	0.66	0.035	0.3	0.00043	COG4430S		conserved hypothetical protein
SAA6008_02492	0.26	0.000014	0.41	0.00018	COG0531E		APC family amino acid-polyamine-organocation transporter
SAA6008_02509	0.24	0.014	0.05	0.00068	COG4408S		conserved hypothetical protein
SAA6008_02510	0.31	0.0016	0.07	0.00015	COG1064R	*fmrO*	rRNA methyltransferase
SAA6008_02511	0.16	0.0082	0.01	0.025	COG0253E	*dapF*	diaminopimelate epimerase
SAA6008_02519	0.64	0.012	0.17	0.048	COG4221R		putative short chain dehydrogenase
SAA6008_02528	0.25	0.01	0.24	0.0069			hypothetical protein
SAA6008_02543	0.65	0.021	0.47	0.00091	COG0789K		transcriptional regulator, MerR family
SAA6008_02561	0.51	0.0039	0.32	0.00084	COG0778C		nitroreductase family protein
SAA6008_02574	0.39	0.0041	0.43	0.0003	COG0824R		conserved hypothetical protein
SAA6008_02595	0.64	0.032	0.6	0.0085			conserved hypothetical protein
SAA6008_02596	0.4	0.001	0.18	0.00031	COG2217P	*copA*	P-ATPase superfamily P-type ATPase copper (Cu2+) transporter
SAA6008_02598	0.4	0.000069	0.43	0.0031	COG1052CHR		D-isomer specific 2-hydroxyacid dehydrogenase family protein
SAA6008_02599	0.49	0.00088	0.29	0.003	COG0436E		N-succinyldiaminopimelate aminotransferase
SAA6008_02615	0.53	0.00063	0.29	0.023	COG4367S		conserved hypothetical protein
SAA6008_02660	0.31	0.00088	0.28	0.0018	COG0531E		APC family amino acid-polyamine-organocation transporter
SAA6008_02661	0.14	0.000087	0.25	0.00016	COG0160E		4-aminobutyrate aminotransferase
SAA6008_02670	0.18	0.0012	0.17	0.0058	COG1012C	*gbsA*	glycine betaine aldehyde dehydrogenase
SAA6008_02671	0.59	0.00088	0.35	0.00052	COG1510K		transcriptional regulators-like protein
SAA6008_02679	0.43	0.035	0.49	0.0075	COG0386O	*gpxA2*	SAA6008_02679 putative glutathione peroxidase
SAA6008_02705	0.36	0.000093	0.27	0.012		*sasF*	LPXTG cell wall surface anchor family protein
SAA6008_02744	0.55	0.0028	0.19	0.00061	COG4720S		putative membrane protein
SAA6008_02753	0.31	0.011	0.17	0.0077	COG2962R	*rarD*	DMT superfamily drug/metabolite transporter
**UPREGULATED**							
SAA6008_00089	4.1	0.0016	2.14	0.016	COG5271R	*spa*	Immunoglobulin G binding protein A precursor
SAA6008_00194	6.78	0.000069	7.03	0.00065	COG1882C	*pflB*	formate C-acetyltransferase
SAA6008_00199	1.93	0.012	5.16	0.0097		*coa*	staphylocoagulase precursor
SAA6008_00233	1.7	0.0013	1.98	0.00056	COG2846D	*scdA*	cell division and morphogenesis-related protein
SAA6008_00235	1.74	0.041	2.23	0.016	COG3279KT	*lytR*	two component transcriptional regulator
SAA6008_00313	1.69	0.025	1.61	0.016	COG2271G	*glpT*	glycerol-3-phosphate transporter
SAA6008_00376	1.54	0.0087	2.4	0.0039	COG0406G		putative phosphoglycerate mutase family protein
SAA6008_00388	1.98	0.038	3.81	0.0036	COG2233F	*pbuX*	xanthine permease
SAA6008_00455	2.21	0.033	2.43	0.0002	COG1009CP	*ndhF*	NADH dehydrogenase subunit L
SAA6008_00456	2.76	0.007	4.03	0.013	COG3002S		conserved hypothetical protein
SAA6008_00478	2.34	0.00051	2.34	0.036	COG1263G		PTS system trehalose-specific IIBC component
SAA6008_00490	1.61	0.000087	2.3	0.013	COG4467S		DNA replication initiation control protein YabA
SAA6008_00494	1.56	0.001	1.57	0.0016	COG0143J	*metS*	methionyl-tRNA synthetase
SAA6008_00525	3.23	0.0014	10.63	0.00018	COG0214H	*pdxS*	pyridoxal biosynthesis lyase
SAA6008_00567	2.86	0.00017	2.05	0.0029	COG0431R	*azo1*	putative FMN-dependent NADPH-azoreductase
SAA6008_00623	13.75	0.00034	4.65	0.04	COG1064R	*adh1*	zinc-binding alcohol dehydrogenase
SAA6008_00627	1.88	0.00069	2.63	0.0043	COG0614P		iron (Fe3+) ABC superfamily ATP binding cassette transporter, binding protein
SAA6008_00680	1.81	0.0021	2.14	0.016	COG0306P	*pitA*	low-affinity inorganic phosphate transporter
SAA6008_00716	1.91	0.0002	2.28	0.00055	COG1299G		PTS transport system, fructose-specific IIABC component
SAA6008_00723	1.52	0.022	2.04	0.00017			putative membrane protein
SAA6008_00724	2.9	0.014	7.88	0.00011			conserved hypothetical secreted protein
SAA6008_00753	1.73	0.0012	2.01	0.028	COG2320S		conserved hypothetical protein
SAA6008_00784	1.59	0.0034	3.12	0.0035	COG1090R		NAD dependent epimerase/dehydratase family protein
SAA6008_00838	1.77	0.00038	3.01	0.00038	COG0582L	*int*	pathogenicity island protein, integrase
SAA6008_00840	1.9	0.00042	1.54	0.002	COG1396K	*hlb*	staphylococcal enterotoxin type I
SAA6008_00882	1.6	0.045	1.77	0.011			conserved hypothetical protein
SAA6008_00886	1.56	0.0028	6.5	0.013	COG3966M	*dltD*	D-alanine lipoteichoic acid and wall teichoic acid esterification secreted protein
SAA6008_00899	2.06	0.00098	4.89	0.0052	COG1320P	*mnhG*	CPA3 family monovalent cation:proton (H+) antiporter-3 subunit G / Na+/H+ antiporter subunit
SAA6008_00913	6.92	0.0002	10.62	0.002	COG0165E	*argH*	argininosuccinate lyase
SAA6008_00914	5.41	0.001	10.61	0.00000075	COG0137E	*argG*	putative argininosuccinate synthase / argininosuccinate synthase
SAA6008_00922	2.24	0.00099	5.87	0.0015	COG0841V		conserved hypothetical membrane protein
SAA6008_00988	3.87	0.00029	3.99	0.01			conserved hypothetical protein
SAA6008_00991	2.7	0.0048	2.2	0.00053	COG2259S		putative DoxX family membrane protein
SAA6008_01023	1.6	0.0025	1.73	0.036	COG1828F	*purS*	phosphoribosylformylglycinamidine synthase
SAA6008_01026	2.08	0.00038	1.9	0.0062	COG0034F	*purF*	phosphoribosylpyrophosphate amidotransferase

SAA6008_01027	2.8	0.00072	2.82	0.00026	COG0150F	*purM*	phosphoribosylaminoimidazole synthetase
SAA6008_01028	2.57	0.0021	2.18	0.00075	COG0299F	*purN*	phosphoribosylglycinamide formyltransferase
SAA6008_01029	3.14	0.000096	1.68	0.042	COG0138F	*purH*	bifunctional phosphoribosylaminoimidazolecarboxamide formyltransferase/IMP cyclohydrolase
SAA6008_01030	2.68	0.000014	2.7	0.0025	COG0151F	*purD*	phosphoribosylamine--glycine ligase
SAA6008_01041	3.21	0.0007	4.77	0.0034	COG1271C		cytochrome ubiquinol oxidase
SAA6008_01043	1.7	0.0016	2.26	0.00018	COG0569P	*trkA*	NAD+ binding potassium transporter
SAA6008_01053	1.69	0.044	7.73	0.02	COG1476K		transcriptional regulator Cro/CI family
SAA6008_01111	4.58	0.0011	5.49	0.0061		*efb*	fibrinogen-binding protein precursor
SAA6008_01112	4.79	0.001	7.5	0.00099		*scc*	fibrinogen-binding protein precursor-related protein
SAA6008_01152	3.16	0.00042	6.98	0.0018	COG2065F	*pyrR*	pyrimidine regulatory protein
SAA6008_01153	2.58	0.0043	10.34	0.0000039	COG2233F	*pyrP*	uracil permease
SAA6008_01154	3.29	0.0029	10.5	0.00053	COG0540F	*pyrB*	aspartate carbamoyltransferase catalytic subunit
SAA6008_01155	2.92	0.0077	8.61	0.00013	COG0044F	*pyrC*	dihydroorotase
SAA6008_01156	2.86	0.039	15.38	0.00000075	COG0505EF	*pyrAA*	carbamoyl-phosphate synthase small subunit
SAA6008_01234	1.83	0.00029	1.97	0.0037	COG0612R		zinc-dependent peptidase, M16 family
SAA6008_01264	4.47	0.00066	3.13	0.012			conserved hypothetical protein
SAA6008_01287	2.1	0.0016	2.97	0.044			conserved hypothetical protein
SAA6008_01318	2.27	0.00053	4.12	0.016	COG0344S	*plsY*	glycerol-3-phosphate acyltransferase PlsY
SAA6008_01321	2.07	0.00003	2.96	0.00084	COG1115E	*alsT*	amino acid carrier protein (sodium/alanine symporter)
SAA6008_01373	1.95	0.00042	1.57	0.023	COG1114E	*braB*	branched-chain amino acid carrier protein
SAA6008_01405	13.76	0.02	4.99	0.014	COG0531E	*frlA*	fructoselysine transporter
SAA6008_01407	3.61	0.0012	3.98	0.018	COG0686E	*ald1*	alanine dehydrogenase
SAA6008_01410	3.37	0.00017	4.1	0.0066	COG3182S	*piuB*	putative iron-regulated membrane protein
SAA6008_01785	1.59	0.007	1.82	0.00081			conserved hypothetical protein
SAA6008_01799	1.86	0.014	1.58	0.0012	COG3481R	*cbf1*	3'-5' exoribonuclease YhaM / cmp-binding-factor 1
SAA6008_01815	3.71	0.0065	11.6	0.016	COG0765E		polar amino acid ABC transporter, inner membrane subunit
SAA6008_01835	6.96	0.000069	4.12	0.015	COG5546S		phage holin
SAA6008_02155	1.74	0.00013	2.41	0.00015	COG2890J		modification methylase HemK family protein
SAA6008_02156	2.51	0.000093	3.3	0.0017	COG0216J	*prfA*	peptide chain release factor RF1 / peptide chain release factor 1
SAA6008_02157	1.77	0.00036	2.62	0.00056	COG1435F	*tdk*	thymidine kinase
SAA6008_02174	1.68	0.006	1.56	0.039	COG0213F	*pdp*	putative pyrimidine-nucleoside phosphorylase / pyrimidine-nucleoside phosphorylase
SAA6008_02336	1.58	0.0061	3.59	0.0017	COG1757C	*nhaC*	putative Na+/H+ antiporter
SAA6008_02342	2.06	0.00017	2.78	0.00013	COG4193G	*lytA*	Mannosyl-glycoprotein endo-beta-N-acetylglucosaminidase
SAA6008_02398	2.76	0.034	2.3	0.0073	COG0577V		hemin ABC superfamily ATP binding cassette transporter, permease component
SAA6008_02432	1.79	0.023	3.07	0.00075	COG2197TK		two component transcriptional regulator, LuxR family
SAA6008_02433	3.5	0.0019	4.01	0.0063	COG4585T	*nreB*	oxygen sensor histidine kinase
SAA6008_02607	2.33	0.00054	6.22	0.011		*isaA*	immunodominant antigen A, transglycosylase IsaA
SAA6008_02751	1.91	0.015	2.26	0.0056	COG4932M	*can*	collagen adhesin domain protein
