# Supplementary material for: Evolution of Multidrug Resistance during Staphylococcus aureus Infection Involves Mutation of the Essential Two Component Regulator WalKR
Source: PLoS Pathog. 2011 Nov 10;7(11):e1002359. doi: 10.1371/journal.ppat.1002359 (PMC3213104; doi:10.1371/journal.ppat.1002359)
Supplement: Table S2 — List of primers used in this study. (DOCX) [file ppat.1002359.s004.docx]

**Table S2**. Primers used in this study

| Primer name | Sequence (5’ → 3’) | Comment |
| --- | --- | --- |
| 1901 | **GGGGACAAGTTTGTACAAAAAAGCA**  **GGCT**GCGCACAATAACAAAAATAG | Used for amplification of *walKR* region and recombination into pKOR1. Contains attB1 sequence at 5’ end (bold). |
| 1908 | **GGGGACCACTTTGTACAAGAAAGCT**  **GGGT**TTATTATTCATCCCAATCACC | Used for amplification of *walKR* region and recombination into pKOR1. Contains attB2 sequence at 5’ end (bold). |
| 1903 | ATTTCCTCCAACAACATGAG | Sequencing primer for *walKR* region. |
| 1905 | AAGAAGCGCAGGCTAATAC | Sequencing primer for *walKR* region. |
| 1906 | AATTTGTTGCCAATGTATCAC | Sequencing primer for *walKR* region. |
| pKOR1insertF | CAGCTGGATGGCAAATAATG | Used to amplify pKOR1 inserts. |
| pKOR1insertR | CTCGGGCCCCAAATAATG | Used to amplify pKOR1 inserts. |
| yycHF | TCCACGTGAAACAAAATCCA | Used for amplification and sequencing of *yycHF*. |
| yycHR | TCATTAATGTGTGAGCGATTGA | Used for amplification and sequencing of *yycHF*. |
| yycIF | CGTTCAAGCTTAGCGAACAA | Used for amplification and sequencing of *yycIF*. |
| yycIR | TATCAAGCGGCTCATCCTTT | Used for amplification and sequencing of *yycIF*. |
| yycJF | GAACCGCATTGTAAAATTAACG | Used for amplification and sequencing of *yycJF*. |
| yycJR | CCATTTCCCACCAACAACAT | Used for amplification and sequencing of *yycJF*. |
| RT_RNAIIIF | TTCACTGTGTCGATAATCCA | Used for *agr* qPCR. |
| RT_RNAIIIR | TGATTTCAATGGCACAAGAT | Used for *agr* qPCR. |
| gyrBF | CCGATTGCTCTAGTAAAAGTCCTG | Used to amplify *gyrB* for RT-PCR. |
| gyrBR | CGTAATGGTAAAATCGCCTGC | Used to amplify *gyrB* for RT-PCR. |
| argHF | ACCTGAAGAGTGGGTTGACG | Used to amplify *argH* gene in RT-PCR |
| argHR | TCGCAAGCATAGTTGCATGT | Used to amplify *argH* gene in RT-PCR. |
| pyrAF | TCCTGAAGCAAATCCAGGAC | Used to amplify *pyrA* gene in RT-PCR. |
| pyrAR | GGCATTGATATGACGCTCCT | Used to amplify *pyrA* gene in RT-PCR. |
| gltBF | TGATGGCGCAGGTATTATGA | Used to amplify *gltB* gene in RT-PCR |
| gltBR | ATAACCCCACGGCATATTCA | Used to amplify *gltB* gene in RT-PCR |
| purFF | TAGCCCAAGAAAGCCCTGTA | Used to amplify purF gene in RT-PCR. |
| purFR | CCCATTTCATATGGCAAACC | Used to amplify *purF* gene in RT-PCR. |
| ureAF | AGTTGCACGGTCGTCGTAAAG | Used to amplify *ureA* gene in RT-PCR |
| ureAR | TCGCGTGCACCTTCTAATAA | Used to amplify *ureA* gene in RT-PCR |
| atlF | CCATGGGGTACGCAATCTAC | Used to amplify *atl* gene in RT-PCR. |
| atlR | CACCATTGTTTGCAGCAACT | Used to amplify *atl* gene in RT-PCR. |
